# Ocular accommodation and wavelength: The effect of longitudinal chromatic aberration on the stimulus–response curve

**DOI:** 10.1167/jov.24.2.11

**Published:** 2024-02-27

**Authors:** Maydel Fernandez-Alonso, Abigail P. Finch, Gordon D. Love, Jenny C. A. Read

**Affiliations:** 1 Biosciences Institute, Newcastle University, Newcastle Upon Tyne, UK; 2 Present address: Translational Sensory and Circadian Neuroscience Group, Max Planck Institute for Biological Cybernetics, Tübingen, Germany; 3 Department of Physics, Durham University, Durham, UK; 4 Department of Computer Sciences, Durham University, Durham, UK; 5 Present address: School of Computing, University of Leeds, Leeds, UK

**Keywords:** accommodation, longitudinal chromatic aberration, stimulus-response curve, pupil size

## Abstract

The longitudinal chromatic aberration (LCA) of the eye creates a chromatic blur on the retina that is an important cue for accommodation. Although this mechanism can work optimally in broadband illuminants such as daylight, it is not clear how the system responds to the narrowband illuminants used by many modern displays. Here, we measured pupil and accommodative responses as well as visual acuity under narrowband light-emitting diode (LED) illuminants of different peak wavelengths. Observers were able to accommodate under narrowband light and compensate for the LCA of the eye, with no difference in the variability of the steady-state accommodation response between narrowband and broadband illuminants. Intriguingly, our subjects compensated more fully for LCA at nearer distances. That is, the difference in accommodation to different wavelengths became larger when the object was placed nearer the observer, causing the slope of the accommodation response curve to become shallower for shorter wavelengths and steeper for longer ones. Within the accommodative range of observers, accommodative errors were small and visual acuity normal. When comparing between illuminants, when accommodation was accurate, visual acuity was worst for blue narrowband light. This cannot be due to the sparser spacing for S-cones, as our stimuli had equal luminance and thus activated LM-cones roughly equally. It is likely because ocular LCA changes more rapidly at shorter wavelength and so the finite spectral bandwidth of LEDs corresponds to a greater dioptric range at shorter wavelengths. This effect disappears for larger accommodative errors, due to the increased depth of focus of the eye.

## Introduction

**Figure 1.**
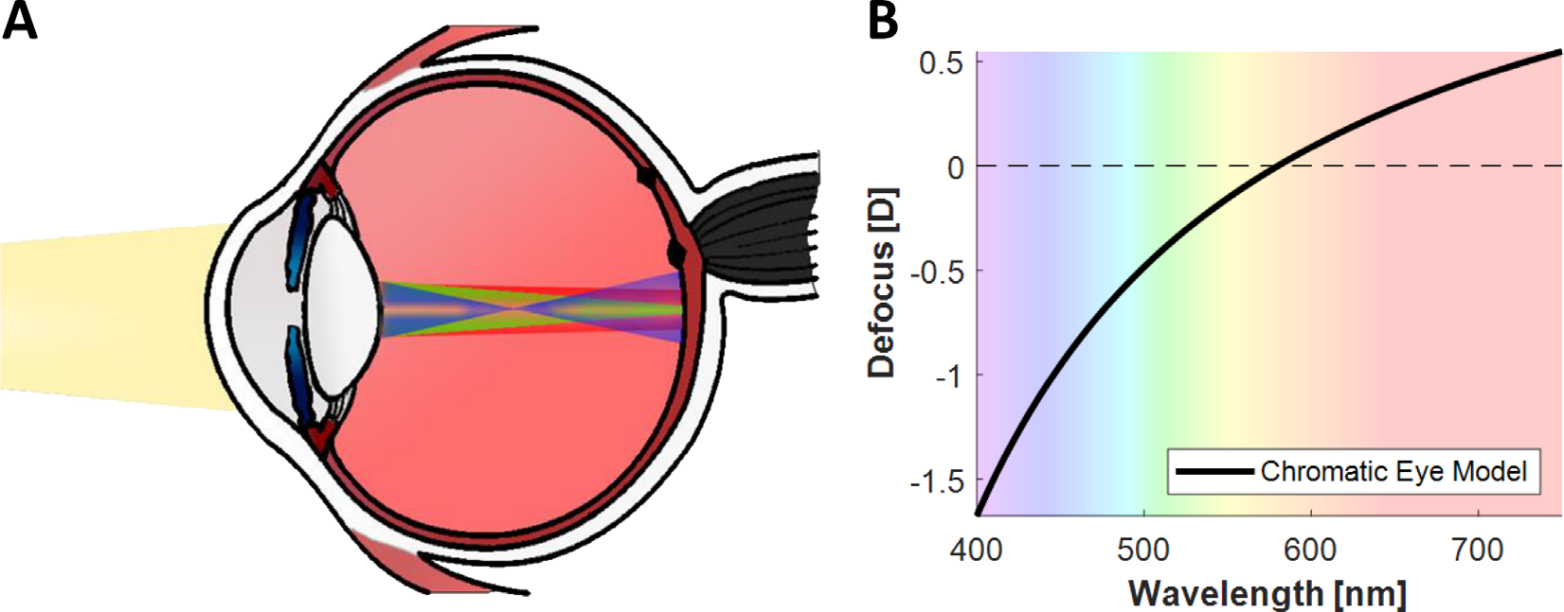
The longitudinal chromatic aberration of the eye. Diagram shows the change in refractive index with wavelength (**A**), and the defocus caused by LCA as a function of wavelength according to the chromatic eye model (**B**). The chromatic eye model specifies the eye's refractive error as *D*(λ) = *p* – *q*/(λ – *c*), where λ is the wavelength of light in micrometers and *D*(λ) is the refractive error in diopters (Thibos et al., 1992). For the three parameters, we took the values used by Marimont and Wandell (1994): *p* = 1.7312, *q* = 0.63346, and *c* = 0.21410; the reference wavelength that is kept in focus is 580 nm.

The purpose of the ocular lens is to adjust the optical power of the eye so as to produce a sharp, in-focus image on the retina. However, its ability to achieve this is affected by the longitudinal chromatic aberration (LCA) of the eye. The refractive index of the eye decreases with an increase in wavelength, such that for a broadband light the shorter wavelengths come into focus in front of the retina and the longer wavelengths behind the retina. The resulting defocus as a function of wavelength is shown in Figure 1B (Thibos, Ye, Zhang, & Bradley, 1992). The total defocus across the entire visible spectrum is approximately 2 diopters (D). This means that the lens is unable to simultaneously optimize ocular power for all visible wavelengths. If green light is in focus, as shown in Figure 1, red and blue light will be out of focus, with positive and negative defocus errors, respectively.

### Role of LCA

Thus, LCA has several implications for visual perception in broadband polychromatic light, such as daylight. Most obviously, image quality can be reduced in polychromatic light compared to monochromatic light (Aggarwala, Kruger, Mathews, & Kruger, 1995; Campbell & Gubisch, 1967). Similarly, contrast sensitivity is greater if chromatic aberration is corrected with achromatizing lenses or reduced by using monochromatic light (Artal, Manzanera, Piers, & Weeber, 2010; Williams et al., 2000; Yoon & Williams, 2002).

However, LCA also implies a greater depth of field in polychromatic light—that is, a greater range of accommodation values for which the image will appear acceptably sharp in the retina, because at least one wavelength is in focus (Campbell, 1957; Campbell & Gubisch, 1967; Marcos, Moreno, & Navarro, 1999). An increased depth of field in polychromatic light has been proposed as a possible explanation for the non-linearity of the human accommodation function. A steady-state error is typically found when accommodation is measured for different distances with white light, and it has been proposed that this could be explained by a change in the component wavelength that is brought into focus at different distances (Ivanoff, 1949). For nearer distances, short-wavelength components would be brought into focus, but, for farther distances, long-wavelength components would be the ones in focus. Due to the increased depth of field, the image formed in the retina would remain acceptably sharp in both conditions. However, evidence already exists against this idea (Bobier, Campbell, & Hinch, 1992; Labhishetty, Cholewiak, Roorda, & Banks, 2021).

In addition to reducing retinal image contrast and increasing the depth of field, LCA can also contribute an odd-error cue to accommodation. In an eye free of aberrations, positive defocus and negative defocus both produce the same effect on the point-spread function at a given wavelength. Thus, in monochromatic light, it is impossible to know whether image contrast will be improved by reducing or increasing ocular power. However, in polychromatic light, one can infer the sign of defocus by comparing the amount of blur at different wavelengths. If red light is blurred more than blue, accommodation should be relaxed, and vice versa. There is evidence that this polychromatic blur serves as an important cue to accommodation (Fincham, 1951; Kruger, Mathews, Aggarwala, & Sanchez, 1993).

### Narrowband primaries

One of the main differences between digital displays and the natural environment is in the spectral distribution of the light they emit or reflect. Whereas daylight is composed of a smooth spectrum and natural objects tend to have broad spectral reflectance functions (Krinov, 1953), most digital displays take advantage of the fact that human vision is trichromatic and make use of only three lights or primaries to show us different images. These primaries (red, green, and blue) give rise to a spectral distribution with multiple narrowband peaks rather than a smooth spectrum, with modern displays increasingly making use of particularly narrowband light sources such as light-emitting diodes (LEDs), organic LEDs (OLEDs), and lasers. As these lights differ significantly from the natural light that the human visual system evolved to accommodate under, it is important to understand how they affect the accommodative response of the eye in order to maximize the quality of the image perceived in these displays.

#### Do narrowband primaries affect the accuracy of accommodation?

The impact of reduced spectral bandwidth and removing LCA on the accuracy of dynamic accommodation responses has been investigated in several studies. Kruger et al. (1993) measured dynamic accommodation responses under broadband and narrowband light while the LCA of the eye was normal, removed, or reversed. They found lower accommodative gain and higher phase lag under narrowband light or when LCA was removed; reversing the sign of LCA severely disrupted the accommodation response. Aggarwala, Kruger, et al. (1995) further explored this by varying spectral bandwidth and found that larger bandwidth increased accommodative gain and decreased phase lag, particularly with broadband white light. In a similar study, these authors found that accommodative gain was higher and phase lag lower when the target was illuminated by white light with LCA intact, compared with monochromatic light or white light with LCA removed (Aggarwala, Nowbotsing, & Kruger, 1995). They also found considerable variability in the ability of subjects to track the monochromatic moving targets, with the authors concluding that narrowband illumination was a poor stimulus for accommodation. The authors suggested that visual displays that used narrowband primaries were likely to reduce the ability of the eye to maintain accurate focus.

On the other hand, studies investigating steady-state accommodation responses have not found evidence that the absence of LCA has a detrimental effect. Bobier et al. (1992) measured the accommodation stimulus–response curve with LCA intact, neutralized, increased, or reversed. They found no differences among any of the conditions tested, with only one participant showing an effect on the reversed LCA condition. When looking at the variability of the steady-state responses, Atchison, Strang, and Stark (2004) found no differences between broadband and narrowband stationary targets, suggesting that focus can be maintained under reduced spectral bandwidth.

The disparity in findings could suggest that LCA may be more crucial for dynamic rather than steady-state accommodation. Kotulak, Morse, and Billock (1995) found evidence in favor of this hypothesis, with their results showing that a broader spectral bandwidth increased the gain of dynamic responses (although no differences in phase lag) but had no effect on steady-state accommodative errors. In contrast, later studies by Kruger, Aggarwala, Bean, and Mathews (1997) showed that steady-state accommodation is affected under reduced spectral bandwidth or reversed LCA, but only when targets are placed away from the tonic state of accommodation. The authors proposed that this was a possible reason for the differing findings in previous studies. Thus, LCA could also be an important cue for steady-state accommodation responses, and the reduced spectral bandwidth of narrowband primaries in a display could impair the accuracy of this response, particularly at near and far distances.

#### Do observers adjust for LCA when accommodating to different primaries?

In addition to a reduced spectral bandwidth, narrowband primaries of different peak wavelengths also impose different accommodative demands due to LCA. When accommodating to monochromatic or narrowband stimuli of different wavelengths, the accommodative response shifts in the direction predicted by LCA—that is, higher accommodation for longer wavelengths and lower for shorter ones (Charman, 1989; Donohoo, 1985; Lovasik & Kergoat, 1988a; Lovasik & Kergoat, 1988b; Seidemann & Schaeffel, 2002), although the magnitude of the dioptric shift has not always been up to the magnitude predicted by the LCA defocus (Donohoo, 1985).

However, these studies have usually tested targets placed at only one or two fixed distances. Few studies to date have looked at the effect of narrowband light of different wavelengths on the accommodation stimulus–response curve. Charman and Tucker (1978) measured accommodation at multiple accommodative demands for white and narrowband illuminants. Most of their seven participants were experienced observers and were able to accommodate under monochromatic light as accurately as under broadband light; however, their one naïve observer initially struggled to accommodate to narrowband targets, requiring further training to perform the task. They also found a dioptric shift in the accommodation responses of participants with wavelength but no difference in their accuracy, such that the stimulus–response curves showed similar lags and leads for all colors tested. They did find, however, a shallower slope in blue light for some observers which they attributed to a combination of a small increase in LCA with accommodation (∼3% per diopter of accommodation) and reduced acuity in blue light.

In a more recent study, Jaskulski, Marín-Franch, Bernal-Molina, and López-Gil (2016) measured the subjective depth of field of seven subjects for targets at three distances (0, 2, and 4 D) when illuminated under broadband and monochromatic light (red, green, and blue). Notably, they conducted these measurements in the paralyzed eye while simulating the higher order aberrations of the accommodated eye using an adaptive optics system. They found that the slopes of the best focus position as a function of accommodative demand were lower than one, but similar between monochromatic and white light.

Although these studies offer crucial insights into the effects of LCA on the stimulus–response curve, their reliance on mostly well-trained observers with experience in accommodation experiments could mean that these findings are not representative of the general population or the average untrained user of visual displays. Previous studies have shown significant intersubject variability in the responses to monochromatic stimuli or to broadband stimuli when LCA has been removed or reversed (Aggarwala, Nowbotsing, et al., 1995) and that naïve observers can struggle to accommodate in monochromatic light (Charman & Tucker, 1978). Furthermore, the approach by Jaskulski et al. (2016) of paralyzing the accommodative and pupil response and relying on subjective reports of perceived blur, might not be a good indication of real accommodative responses with natural pupil sizes.

### The present study

The literature reviewed so far shows that there are not yet clear answers to the two questions posed above. In addition to being of interest scientifically, these questions are important given the increasing use of narrowband primaries in modern visual displays. Any effect on the accuracy or precision of accommodation could lower image quality and increase the risk of visual fatigue. Thus, we aimed to address both questions together, using a larger sample with a greater proportion of untrained observers. Furthermore, we allowed the accommodation and pupil size of observers to vary freely to more closely match a real-life scenario of subjects viewing images in a digital display with narrowband primaries. Finally, we also concurrently measured visual acuity using a staircase procedure to explore the impact that any difference in accommodation to narrowband stimuli might have on the ability of subjects to resolve small targets when compared to accommodation in broadband light where LCA is available as a cue.

## Methods

In Experiments 1 and 2, the accommodation function was sampled by changing the physical distance of the stimuli, with the angular size of the diffuser changing concurrently in Experiment 1 and being kept constant in Experiment 2. In Experiment 3, the accommodation function was measured by using trial lenses to simulate a larger range of distances, and the visual acuity of participants was measured concurrently. Experiments 1 and 2 used the same apparatus and are described together, but Experiment 3 is described separately where necessary.

### Participants

Participants were recruited from students, staff, and the external pool of participants of the Biosciences Institute of Newcastle University for Experiments 1 and 2 and only from students and staff of the Biosciences Institute for Experiment 3. The study adhered to the tenets of the Declaration of Helsinki and was approved by the Newcastle University Faculty of Medical Sciences Research Ethics Committee. Participants read the information sheet and signed the consent form at the start of their first experimental session.

In Experiments 1 and 2, only participants that did not require visual correction (i.e., spectacles or contact lenses) to read or perform other daily activities were selected. The mean visual acuity of the sample was 0.03 logarithm of the minimum angle of resolution (logMAR) with a range between –0.1 and 0.23 logMAR. This means that the smallest characters they could read had a stroke width of 1.1 arcmin on average, with a range in the sample between 0.8 and 1.7 arcmin. In Experiment 3, two of the 10 participants normally used spectacles to read or perform activities at near distances (with corrections of approximately –0.7 D and –2.5 D) but performed the experiment without them, as wearing them would change the intended accommodative demands. Table 1 shows the breakdown of the included sample for each individual experiment.

**Table 1.** Summary of protocol for Experiments 1, 2, and 3.

	Experiment 1	Experiment 2	Experiment 3
Participants, *n*	8	9	10
Age (y), mean ± SD	26.5 ± 2.5	25.9 ± 2.5	29.5 ± 2.4
Females/males, *n*	4/4	4/5	6/4
Stimulus	Maltese cross on diffuser illuminated by LEDs		AMOLED screen with Landolt C targets
Accommodative demand varied using	Physical distance (33 cm to 2 m)		Trial lenses (–7 D to 2 D in steps of 1 D with a physical distance of 1 m)
Accommodative demands used	Six (0.5–3 D in steps of 0.5 D)		Ten (–1 D to 6.5 D after correcting for lens offset)
Illuminants used	Six (441 nm, 460 nm, 527 nm, 588 nm, 661 nm, D65)		Four (459 nm, 528 nm, 610 nm, RGB)
Stimulus size	Physical size fixed (Maltese cross = 5 × 5 cm), angular size varying (1.4°–8.6°)	Angular size fixed (Maltese cross 1.5° × 1.5°), physical size varying (0.9–5.2 cm)	Physical and angular sizes varying to measure acuity (0.08° × 0.08° at 0 logMAR)
Each block consists of	Six stimulus presentations (one for each of 6 illuminants, random order)		4 × 24-trial staircases (one for each of four illuminants, random order)
Duration of each stimulus presentation	8 s	3 s	Until completion of the 24-trial staircase (typically >20 s)
Duration after stimulus onset excluded from analysis	1.5 s	1.5 s	2 s
Distinct blocks, *n*	Six (one for each accommodative demand)		Ten (one for each accommodative demand)
Number of times each distinct block is repeated	≥5	≥12	3

### Apparatus for Experiments 1 and 2

The stimulus consisted of a Maltese cross printed on a transparent film and placed on top of a diffuser, which was mounted on a box containing six LEDs and centered to the right eye. The box was placed on a 2.5-m-long rail positioned at the height of the participant’s eyes, which allowed us to quickly change the physical distance of the stimulus between blocks of trials. The stimulus was presented at six distances between 3 D and 0.5 D in steps of 0.5 D (corresponding to metric distances of 33.3 cm, 40 cm, 50 cm, 66.7 cm, 100 cm, and 200 cm).

For Experiment 1, we kept constant the physical size of the diffuser (8.5 × 8.5 cm) and the Maltese cross (5 × 5 cm) across the different distances, thus changing its angular size. The angular size of the diffuser thus changed between 14.5° and 2.4° in steps of 2.4° for the different distances, and the angular size of the Maltese cross changed between 8.6° and 1.4° in steps of 1.4°, potentially providing a non-accommodative cue to distance. For Experiment 2, we kept the angular size of the diffuser and the Maltese cross constant across the different distances at 2.5° and 1.5°, respectively, in order to determine whether this cue affected results.

**Figure 2.**
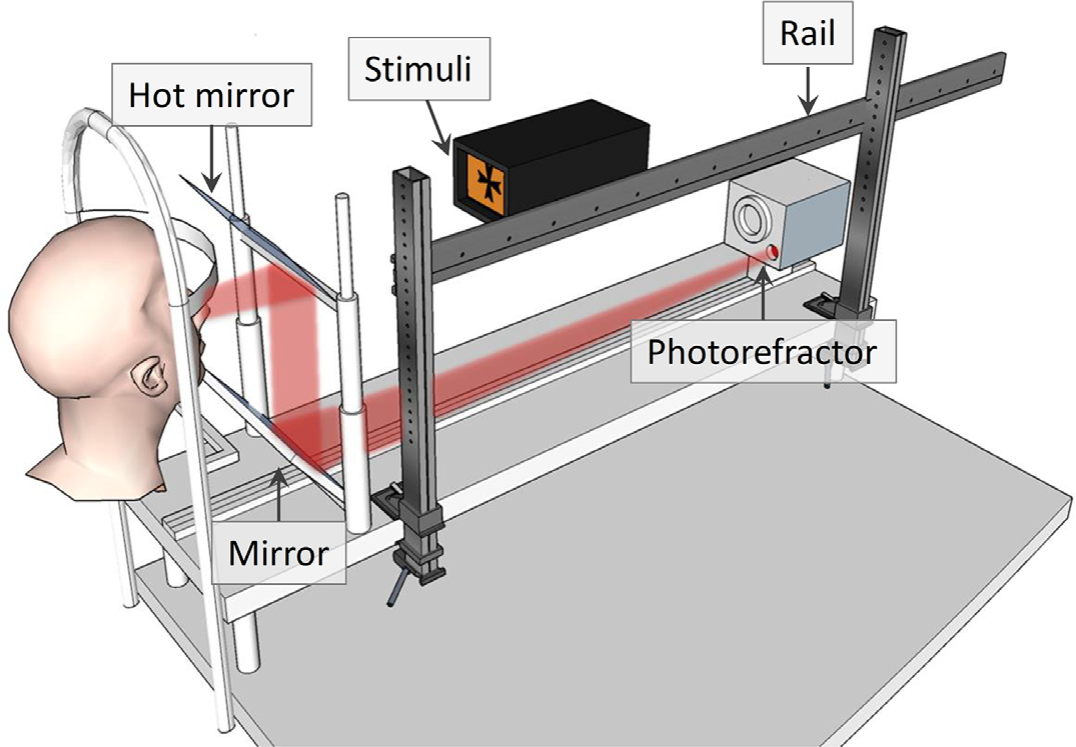
Diagram of the experimental setup for Experiments 1 and 2. The stimuli consist of a black Maltese cross on a bright background formed by a diffuser back-illuminated by LEDs. The color of the background varies depending on which LEDs are used. The physical distance of the stimuli is varied by moving them along a rail. The observer's accommodative state is monitored using a photorefractor, which views the observer's eyes via a hot mirror that transmits visible light while reflecting infrared.

The refractive state of the eye and the pupil diameter was measured dynamically at 50 Hz using a photorefractor with pupillometry capabilities (PowerRef 3; Plusoptix, Nuremberg, Germany). This was calibrated for each subject individually as described in the Supplementary Materials (see Supplementary Figure S1). Two Arduino Uno boards (Arduino, Ivrea, Italy) controlled the stimuli and were connected to the photorefractor to synchronize the recordings with the stimuli. A representation of the experimental setup is shown in Figure 2.

**Figure 3.**
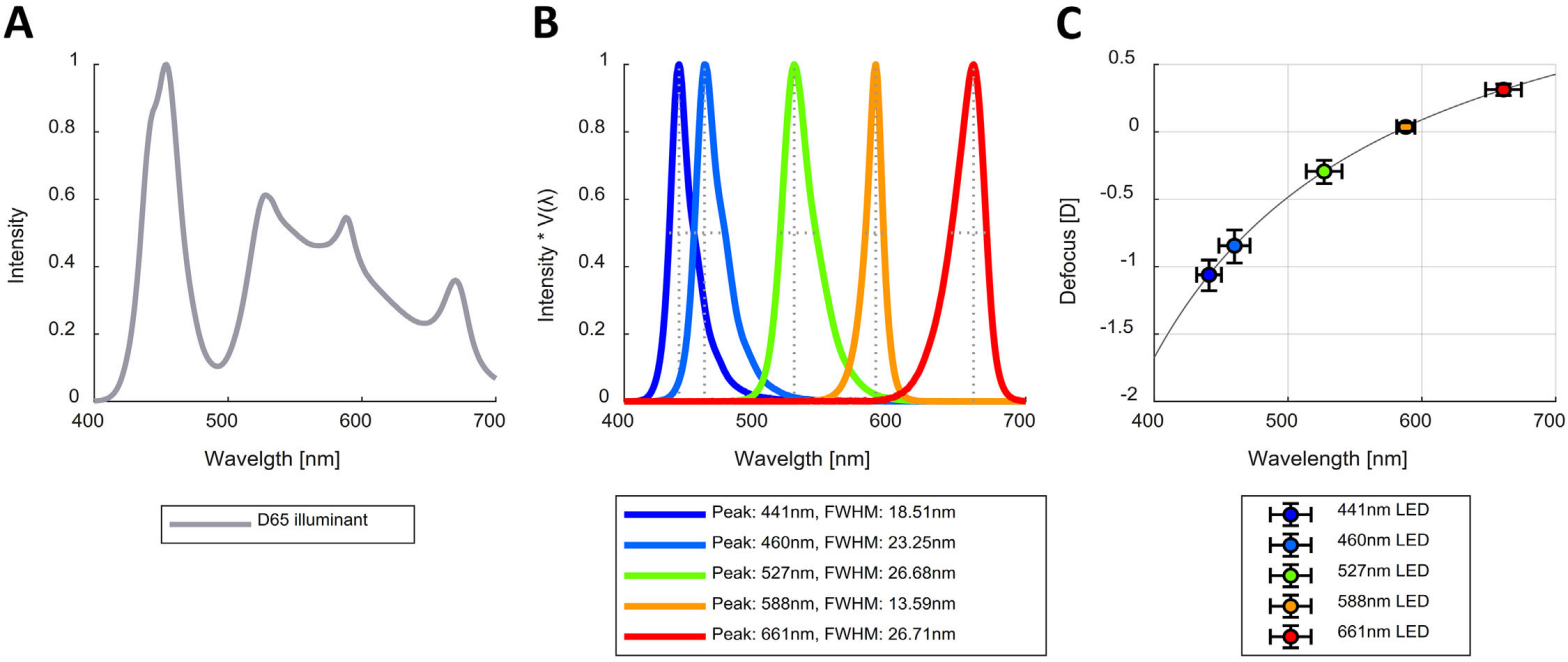
Normalized spectral distributions of the D65 broadband illuminant (**A**) and the narrowband LEDs (**B**) and the defocus caused by LCA for the peak wavelengths of the LEDs (**C**), with horizontal error bars representing the FWHM and vertical error bars the corresponding spread in defocus as predicted by the chromatic eye model (Thibos et al., 1992).

The different spectra were created using six LEDs, five of which were narrowband and one white LED, with the latter being combined with the narrowband LEDs to create a broadband spectral distribution that approximated a D65 illuminant (see Figure 3A). A driver circuit was built for each of the LEDs, and their luminance was controlled through pulse-width modulation from the Arduino Uno boards (at a frequency of 980 Hz). The circuit was designed such that the luminance of the LEDs varied minimally over time by increasing the resistance and decreasing the current through each LED. During the first 10 seconds after each LED was turned on, the luminance remained constant for all LEDs except the red one, for which luminance decreased by ∼0.7 cd/m^2^. Radiance measurements of the LEDs were taken with a CS-2000 Spectroradiometer (Konica Minolta, Tokyo, Japan) at different duty cycles and over time. We multiplied each radiance spectral distribution by the CIE physiologically relevant luminous efficiency function *V*(λ) (Stockman, Jägle, Pirzer, & Sharpe, 2008) to obtain the peak wavelength and full width at half maximum (FWHM) (see Figure 3B). We integrated these to obtain the luminance, which we confirmed was a linear function of duty cycle for each LED. During the experiment, the luminance of all stimuli was kept constant at 10 cd/m^2^, which was the maximum achievable for the red LED.

### Apparatus for Experiment 3

The stimulus consisted of different Landolt C figures that were presented in an active-matrix OLED (AMOLED) screen placed at a fixed distance of 1 m (1 D). The screen had a size of 6.84 × 12.2 cm and a resolution of 1080 × 1920 pixels and was from a OnePlus 3T mobile phone device (OnePlus, Shenzhen, China).

**Figure 4.**
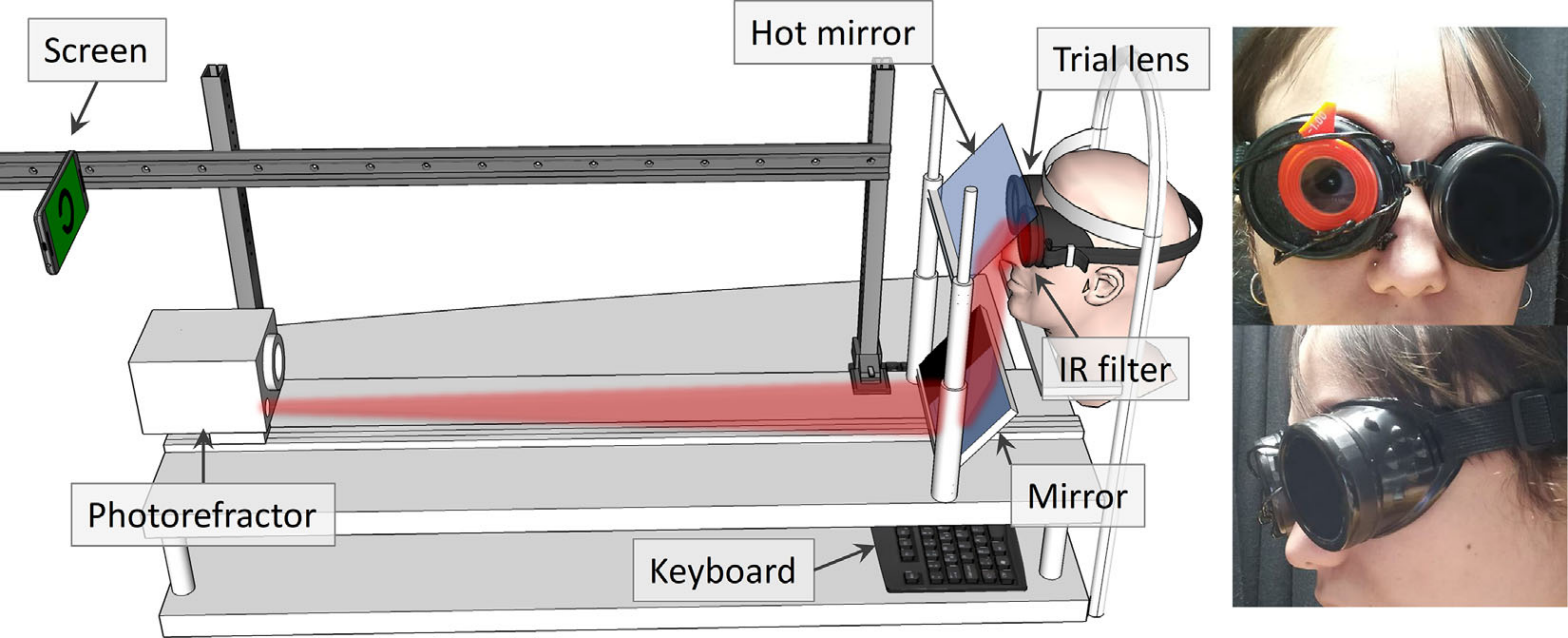
Representation and photographs of the experimental setup for Experiment 3. The setup is similar to that shown in Figure 2, except now the stimuli are presented on an AMOLED screen at a fixed distance of 1 meter. The observer views the stimuli monocularly through a lens placed over their right eye. The left eye is covered by a filter that blocks visible light while allowing the photorefractometer to monitor the refractive state and pupil size using infrared.

To simulate the defocus caused by viewing the stimuli at different distances, nine trial lenses were used with powers that ranged from +2 D to –7 D in steps of 1 D. The stimuli were viewed through the lenses, which were placed over the right eye in light-tight goggles. The left eye was covered by a 720-nm infrared filter that occluded the visual stimuli while allowing the refractive state and pupil diameter of the eye to be measured by the PowerRef 3 photorefractor. A graphical representation and photographs of the experimental setup are shown in Figure 4.

**Figure 5.**
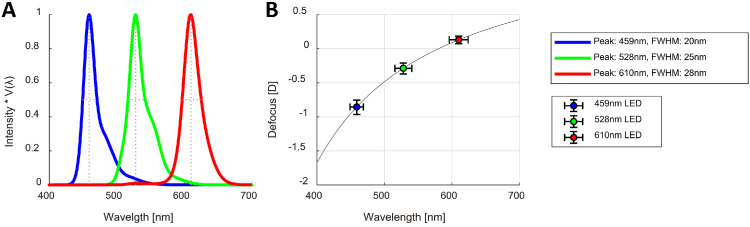
Normalized spectral distributions of the screen LED primaries (**A**) and the defocus caused by LCA for their peak wavelength (**B**), with horizontal error bars representing the FWHM and vertical error bars the corresponding spread in defocus.

The AMOLED screen and experimental routine were controlled from a computer running MATLAB (MathWorks, Natick, MA), which was also connected to the photorefractor to synchronize the stimuli being presented with the recordings. The Landolt C figures were dynamically created using the Psychophysics Toolbox (Kleiner et al., 2007). Figure 5 shows the spectral distributions of the screen primaries, as well as the defocus caused by the LCA of the eye for their peak wavelengths (Thibos et al., 1992).

Radiance measurements of the screen primaries were taken with the CS-2000 Spectroradiometer at different intensities and over time. The peak wavelength and luminance of the LEDs were calculated using the CIE physiologically relevant luminous efficiency function (Stockman et al., 2008). During the experiment, the three primaries of the screen were used at a fixed luminance of 15 cd/m^2^ when used on their own to give narrowband illumination; when they were combined to create a broadband illumination, each primary was given a luminance of 5 cd/m^2^ for the same total luminance of 15 cd/m^2^. This luminance level was chosen because the minimum luminance achievable with one of the primaries was 5 cd/m^2^.

### Design and procedure for Experiments 1 and 2

Each participant’s visual acuity was measured at near and far distances using a Snellen chart and a logMAR test, respectively. All participants had a visual acuity of 0.25 logMAR or better without the need for spectacle or contact lenses; that is, they could read characters that were smaller than 8.9 arcmin wide with a stroke width of 1.8 arcmin. The photorefractor calibration procedure was then performed.

During the experiment, the participant's left eye was covered with an eyepatch, and they sat with their head placed on a chinrest. The participant was instructed to fixate on the stimulus presented and to keep it in focus with as much effort as if they were reading a book. A button placed next to the participant allowed them to pause the task at any time, and frequent breaks were given throughout the experiment.

The distance of the stimuli was varied between experimental blocks, with the order of the distances being randomized among the participants. In Experiment 1 the size of the diffuser and fixation was kept constant, whereas in Experiment 2 it was changed according to the distance of the target to keep a constant angular size. Within each experimental block, the target was illuminated by the five narrowband illuminants and the broadband illuminant, with their order being randomized. In Experiment 1, each illuminant was presented for 8 seconds and repeated at least five times at each of the six distances for a total of 180 trials. In Experiment 2, each illuminant was presented for 3 seconds and repeated 12 times each at each of the six distances, for a total of 432 trials. Between trials, the target was illuminated in both experiments with the orange (588-nm) LED to keep a constant luminance adaptation and to start at a relatively similar accommodation value before the target stimuli were presented. Both experiments took approximately 1 hour to complete.

### Design and procedure for Experiment 3

The photorefractor calibration procedure was performed, and the participant was then given instructions for the visual acuity task. During the experiment, participants sat with their head placed on a chinrest while wearing a pair of light-tight goggles that had an infrared filter over the left eye and allowed us to place different trial lenses over the right eye. Frequent breaks were given between experimental blocks, and participants could pause the experiment at any time.

To measure visual acuity, we used a four-alternative forced-choice (4AFC) task with a best PEST staircase procedure of 24 trials (Kingdom & Prins, 2016). Each Landolt C was presented until the participant gave an answer, and the entire staircase procedure took between 20 and 30 seconds to complete. The background of the Landolt C targets was varied for each staircase according to the four illuminants used in the experiment (three narrowband and one broadband). The order of the illuminant was randomized within each experimental block, and a break of 5 seconds was given between each block where no stimuli was presented. For each experimental block, a different trial lens was placed in front of the participant's right eye to add different values of defocus to the stimulus, and the order of the lenses was randomized among the participants.

The distance of the stimuli (in diopters) was calculated as a function of the physical distance of the screen in diopters (*P_scrn_*), the power of the different lenses placed in front of the eye (*P_lens_*), and the distance from the eye to the lens (*x_lens_*; 3 cm) such that:

$$\begin{array}{c} P_{scrn}^{'}=P_{scrn}\frac{1+P_{lens}\left(x_{lens}-P_{scrn}^{-1}\right)\;}{1+P_{scrn}P_{lens}\left(x_{lens}-P_{scrn}^{-1}\right)x_{lens}\;}. \end{array}$$

Furthermore, the visual acuity thresholds obtained in degrees of visual angle were corrected for the small magnification the lenses produced, which was calculated as:

$$\begin{array}{c} \frac{\theta_{scrn}^{'}}{\theta_{scrn}}=\frac{1}{1+P_{scrn}P_{lens}\left(x_{lens}-P_{scrn}^{-1}\right)x_{lens}}. \end{array}$$

The corrected thresholds in degrees of visual angle were then transformed to logMAR units by converting the values into minutes of visual angle and calculating the base-10 logarithm.

### Data processing and analysis

To analyze the refractive and pupil size recordings, the data points where the pupil was not found were identified as blinks and excluded, as well as 60 ms before and 120 ms after each blink. Blinks would on occasion cause big spikes in the refractive data; thus, any data points where reported refraction was greater than 25 D were also excluded. To allow time for the participants to accommodate, the first 1500 ms of refractive and pupil size data in each trial were excluded from further analysis in Experiments 1 and 2. Similarly, the first 2000 ms of data in each trial were excluded in Experiment 3. Finally, any trial with less than 1000 ms of measurements in Experiments 1 and 2 or 2000 ms of measurements in Experiment 3 were excluded, as well. The calibration correction factor obtained for each participant was then applied to the refraction data, and the median accommodation and pupil size were obtained for each trial.

To perform the analysis on the slopes of the accommodation function, we first determined the linear portion of the accommodation response curve. For this, we calculated the gradient of the accommodation response for each illuminant at each distance, as well as the median gradient; at any distances where the gradient decreased by 50% or more when compared to the median, the response was deemed to be saturated. These results were visually inspected, and some manual corrections were performed, although they mostly agreed well with the visual evaluation of the experimenter.

For the slope and within-trial response variability analyses, the data of Experiment 3 were divided into smaller subsets to improve the fit results. The trials in this experiment had a duration of between 20 and 30 seconds, so each one was divided in equal subsets of at least 5 seconds of duration. For all other analyses, the data were not divided.

Several linear mixed models were used to analyze the effects of distance and illuminant on the slope of the accommodation function, the effects of distance on the difference in accommodation to different wavelengths, the effects of illuminant and accommodation on response variability, the effects of the effects of accommodation on pupil diameter, and the effects of accommodative error on visual acuity. In all cases, we used the maximal random-effects structure without convergence issues. All models were fitted with the maximum likelihood estimation method, and all fits and corresponding residuals were visually inspected to verify that all assumptions were met. For the slope analyses, the median refraction data within each experiment were weighted by the number of valid measurements obtained within each trial as a proportion of the total number of measurements possible. That is, trials where fewer refractive measurements were obtained due to blinking or other factors were assigned a lower weight in the model fits.

The data processing and most of the model fits were performed using MATLAB, and the model fits on visual acuity and the post hoc analyses were done using R (R Foundation for Statistical Computing, Vienna, Austria), particularly the lme4 library (Bates et al., 2023) and the emmeans library (Lenth et al., 2023). Data are available at https://doi.org/10.25405/data.ncl.23531250, and the MATLAB analysis code is available at https://doi.org/10.25405/data.ncl.23522934.

## Results

Results were generally similar for all experiments; that is, the differences in experimental parameters, summarized in Table 1, did not affect the outcome. In the following sections, we thus present results from all three experiments together, except visual acuity, which was measured only in Experiment 3.

**Figure 6.**
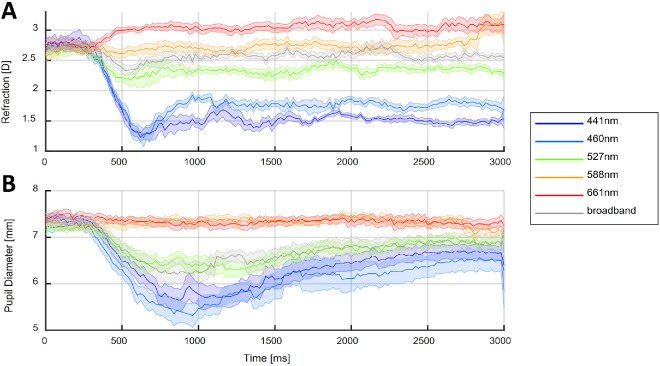
Average accommodation response of subject 2 to different illuminants presented at 33.3 cm (3 D) and repeated 12 times each (Experiment 2). (**A**) The mean refractive state of the eye; (**B**) the mean pupil diameter as a function of time. The continuous lines represent the mean, and the shaded areas represent the standard error of the mean. The time point of 0 ms represents the start of the trials, when the illuminant changed from the 588-nm (orange) to the corresponding illuminant as indicated by the legend. This figure was generated by MATLAB script fit_z_accTraces_e1.m in the code repository.

Figure 6 shows a typical accommodative response for the different illuminants used in Experiments 1 and 2. As shown, a change in the refractive state of the eye occurs after approximately 300 ms from stimuli presentation, alongside pupil constriction for some of the illuminants presented. After 1000 ms, the refractive state of the eye remains relatively constant but the pupil size slowly increases. For subsequent analysis, we use the steady-state response defined as the median value after the initial exclusion period of 1500 ms (see Methods).

### Effects of LCA on the accommodation response curve

**Figure 7.**
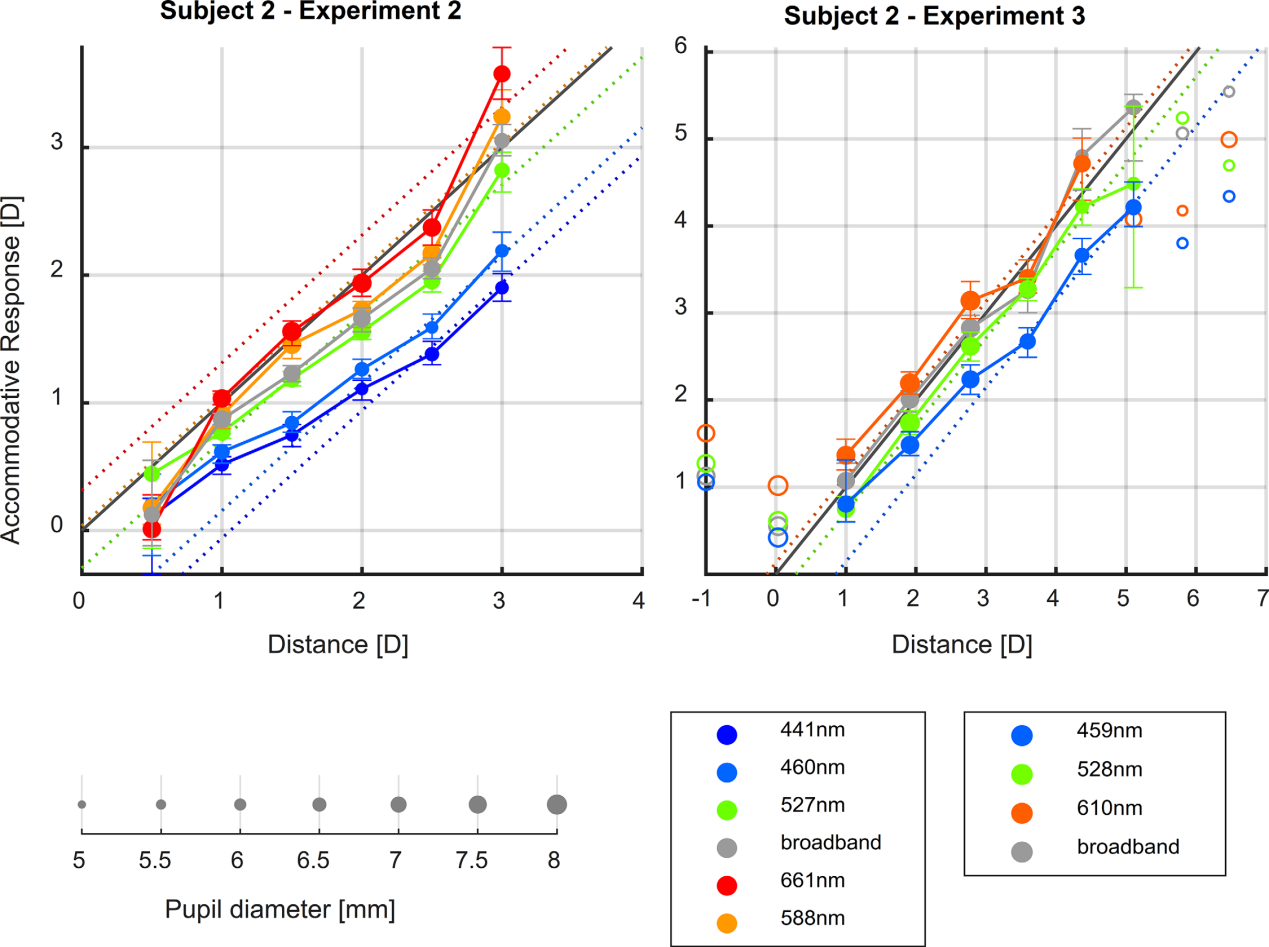
Stimulus–response function for one example participant. Median accommodative response (i.e., ocular refraction) is plotted as a function of the stimulus distance, both in diopters, for the different illuminants. The error bars represent the 25th and 75th percentiles of the response. Filled symbols linked with lines show points classified as being on the linear portion of the stimulus–response curve; empty symbols indicate points classified as outside the range of accommodation and thus not used for fitting. Symbol size represents median pupil diameter.

An example plot of steady-state response is shown in Figure 7 (same subject as Figure 6; plots for all subjects and experiments are shown in Supplementary Figures S2–S4). Figure 7 shows the median response for the different illuminants. For this subject, the farthest distance they can accommodate to is around 0.5 D, and the nearest distance is approximately 5.5 D. These near and far points determine the accommodative range of the subject—that is, the array of distances over which the eye can adjust its optical power to bring an object into focus. Within the accommodative range of the subject, the response for a given illuminant is a quasilinear function of distance, with the absolute value of accommodation changing in accordance with the defocus caused by LCA for each illuminant (i.e., at the same distance, observers accommodate less for shorter wavelengths and more for longer ones). An interesting feature of the data, found in most subjects, is that the slope of the response seemed to change for each illuminant, with a shallower slope observed for shorter wavelengths and a steeper slope for longer ones.

To quantify this effect, we first determined the linear portion of the stimulus–response curve. We defined this as the subject's accommodative range and excluded the stimuli that fell outside it. Further details about this procedure are given in the Supplementary Material. The accommodation response curves of individual participants for each illuminant in the three experiments are also provided in the online data repository.

We fitted linear mixed-effects models, as these allow us to obtain slope and intercept estimates for each illuminant and to account for individual differences among observers. The fits were performed on the median accommodation response and only over the linear portion of the accommodation response curve. Three participants of Experiment 3 (subjects 15, 21, and 22) were excluded from this analysis, as their response curves for most illuminants were only linear over two or three distances.

For each experiment, the linear mixed models were fitted with predictors of distance in diopters, illuminant, and their interaction, as well as random intercepts and slopes of participants (i.e., the effect of distance, illuminant, and their interaction was allowed to vary randomly among observers). Illuminant was used as a categorical predictor because the broadband illuminant with no peak wavelength was included and because the change in slope with peak wavelength for the narrowband illuminants might not be linear (because the defocus caused by LCA as a function of wavelength is not linear). These models were compared in each case with a simpler model that contained no interaction term between distance and illuminant, so the effect of wavelength on accommodation would be constant regardless of distance and the slope for all illuminants would be the same. Results from the likelihood ratio test (LRT) and the Akaike information criterion (AIC) comparison indicated that the model with the interaction term fitted the data better and had greater predictive power in all cases: for Experiment 1, χ^2^ (55) = 177.6, *p* < 0.001, ΔAIC= 67.6; for Experiment 2, χ^2^ (55) = 265.8, *p* < 0.001, ΔAIC= 155.8; and for Experiment 3, χ^2^ (24) = 146.92, *p* < 0.001, ΔAIC= 98.9. This finding means that, in all three experiments, a model that included an interaction term between illuminant and distance (i.e., where we allowed both the intercept and the slope to vary for each illuminant) fitted the data better than a simpler model without the interaction term (i.e., where the intercept but not the slope varied for each illuminant).

**Figure 8.**
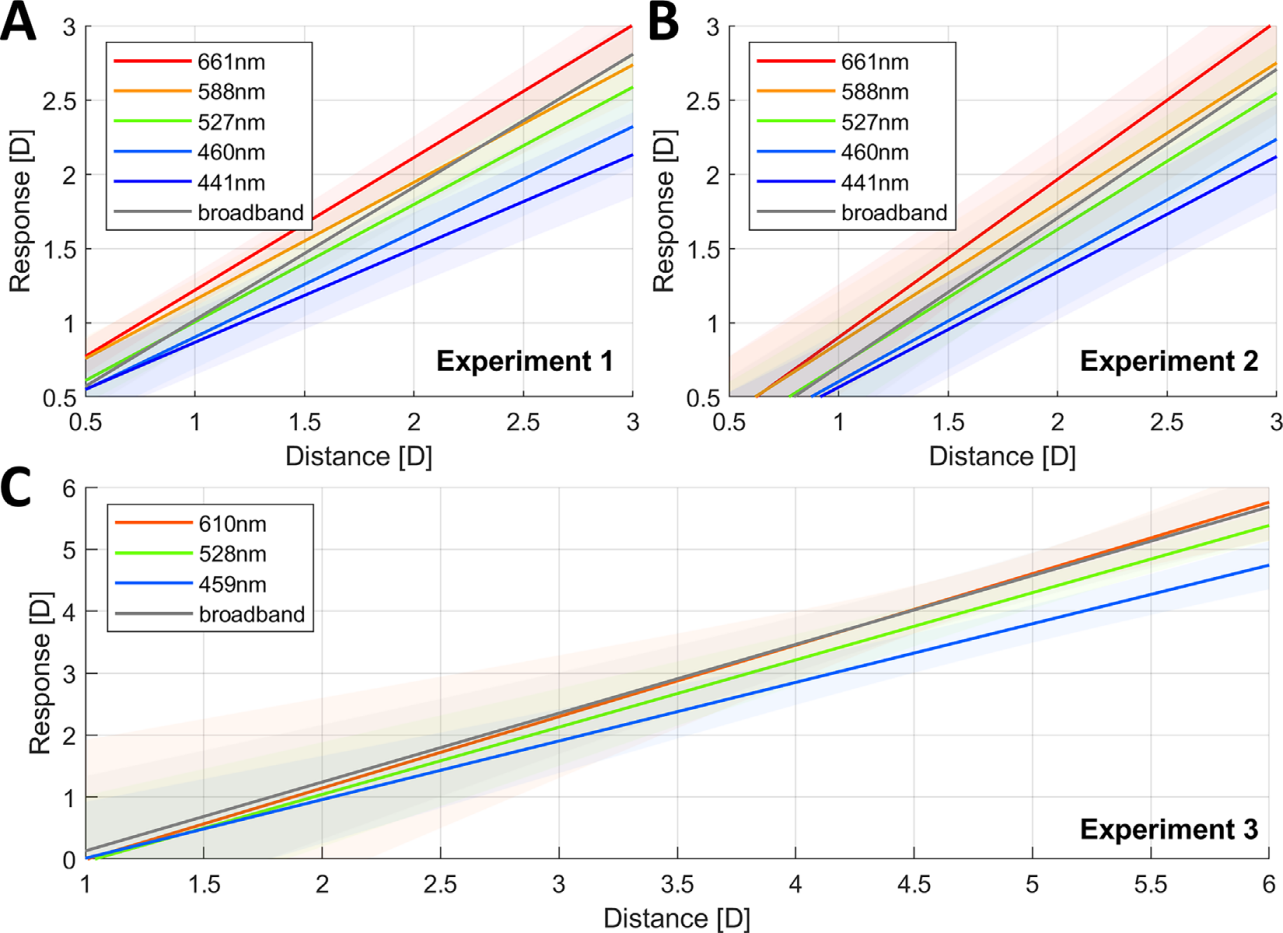
Estimated accommodative response as a function of distance for the linear portion of the accommodation response curve of all participants in each experiment. The continuous line represents the estimated response, and the shaded areas the 95% CIs. The different colors represent the different illuminants used. This figure can be generated with fig_b_oneLMM_123_wDistance_accResp.m in the code repository, and it uses the shadedErrorBar function by Campbell (2023).

The results of the linear mixed models are illustrated in Figure 8 and given in full in Supplementary Table S1. The individual slopes and intercepts estimated for each subject (obtained from the estimated coefficients of the random effects of the model) are presented in Supplementary Table S3. The results show, as illustrated in Figure 8, that the slope of the accommodation response curve for narrowband illuminants becomes shallower as the peak wavelength decreases. Thus, the accommodation responses to narrowband illuminants are mostly similar at optical infinity, but as the stimulus nears the observer the difference in accommodation to different wavelengths increases in correspondence with the defocus caused by LCA. This results in steeper slopes for longer wavelength illuminants and shallower slopes for shorter peak wavelengths.

**Figure 9.**
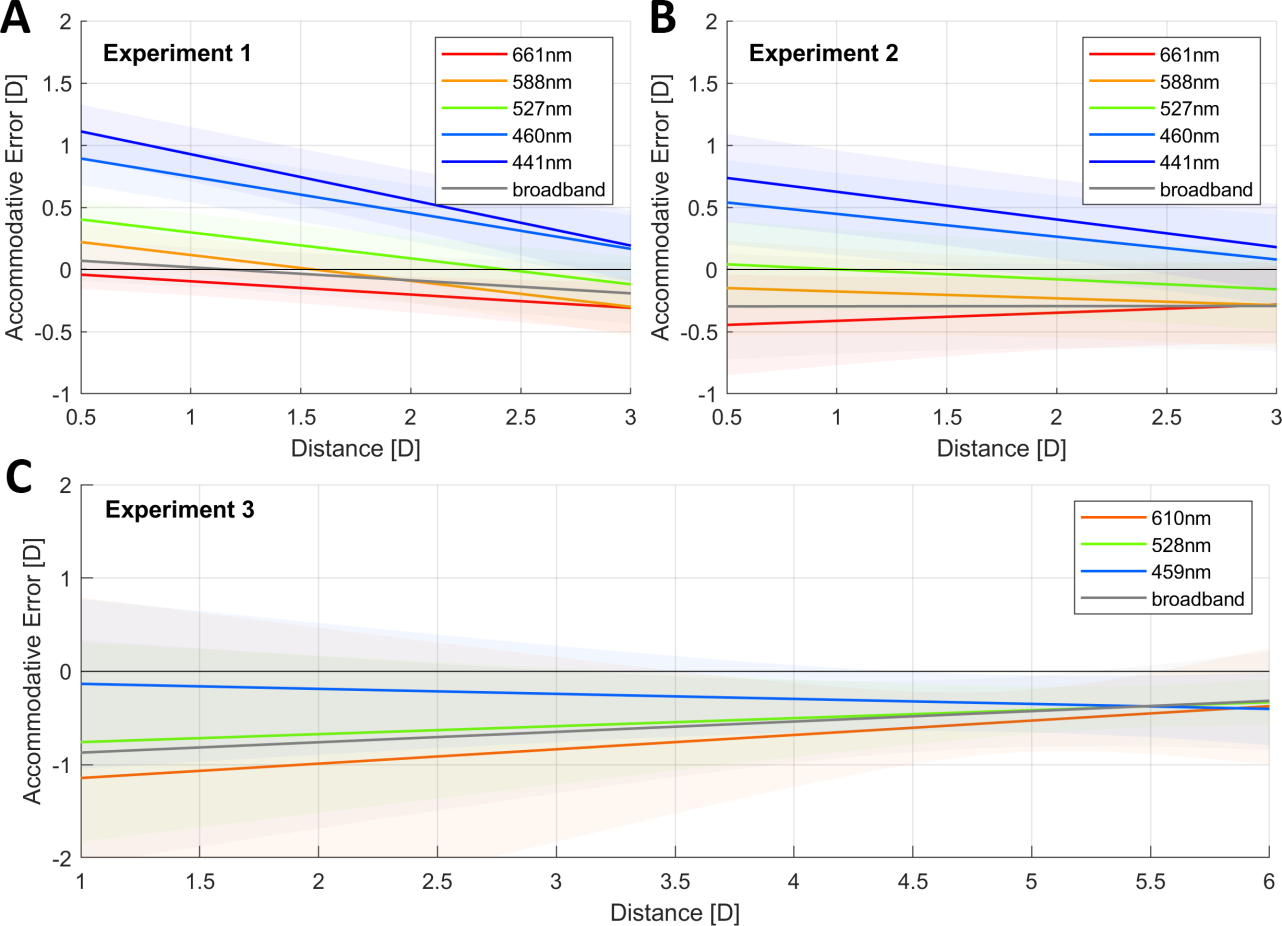
Accommodative error (response–demand) as a function of distance for different wavelengths, as predicted by the linear mixed-effects models fitted to the data of each experiment. The continuous lines of different colors represent the predicted responses for different illuminants, as indicated by the legend, and the shaded regions represent the 95% CIs. Note the wider range of the horizontal axis for Experiment 3. This figure can be generated with fig_b_LMM_123_wDistance_accError.m in the code repository, and it uses the shadedErrorBar function by Campbell (2023).

To illustrate how the change in slope for different illuminants affects the lags and leads of the linear portion of the accommodation response curve, Figure 9 shows the accommodative error as a function of distance for each illuminant. We calculated the accommodative error by subtracting the demand from the predicted response; thus, a negative error indicates that the eye is underaccommodating or focusing farther away than where the target is (accommodative lag), and a positive error indicates that the eye is focusing nearer than the stimulus (accommodative lead). The accommodative demand is given by the distance of the stimuli and the defocus caused by LCA for the peak wavelength of the narrowband illuminant, which we calculated following the chromatic eye model by Thibos et al. (1992). As illustrated, the increased difference in the accommodative response to different wavelengths as the stimulus is placed nearer corresponds to the change in demand caused by LCA. In other words, participants are increasingly compensating for LCA as the target is placed at nearer distances, causing the accommodative error to become both smaller and less dependent on wavelength. Furthermore, accommodation is more accurate for middle wavelengths over most distances, with a tendency to overaccommodate for shorter wavelengths and underaccommodate for longer ones, and the accommodative errors for all wavelengths and in all three experiments seem to approach a small negative value of approximately –0.5 D rather than zero, indicating a small accommodative lag at nearer distances. This lag may in fact maximize image quality due to factors such as spherical aberration (Labhishetty et al., 2021).

### Effects of distance on the accommodation response to different wavelengths

As shown previously, the extent to which participants change their accommodative responses under illuminants of different wavelengths to compensate for the LCA of the eye changes with the distance of the stimulus. To determine the rate of this change, we fitted a linear mixed model to the relative differences in accommodation between wavelengths, using data from all three experiments. The model fixed effects were distance in diopters and the LCA defocus predicted by the chromatic eye model, plus their interaction, and the random effects were the slope and intercept of participant.

The accommodation responses were first centered around the response to the green illuminant (527 nm in Experiments 1 and 2, 528 nm in Experiment 3) for each subject at each distance, and the defocus predicted by the chromatic eye model was set to be zero at 527.5 nm. That is, we are *assuming* that accommodation is such that the green illuminant is in focus for each subject at each distance, and we are examining how accommodation varies with wavelength around this, allowing for the fact that the effect of wavelength may vary with distance.

In the previous analysis, illuminant was being treated as a categorical predictor and no assumptions were being made about how it affected accommodation, but here we are using the LCA defocus predicted by the chromatic eye model (Thibos et al., 1992) for the peak wavelength of the narrowband illuminants and examining how well it predicts the differences in accommodation among illuminants. A slope of 1 for this relationship would indicate that observers are fully compensating for the LCA of the eye when accommodating to the narrowband illuminants, whereas a slope of 0 would indicate that there are no differences in the accommodation response to different wavelengths (i.e., they are not correcting for the defocus caused by LCA). Furthermore, we are also exploring here how this relationship between LCA defocus and the difference in accommodation to different wavelengths changes as a function of distance. Based on the previous results presented thus far, we would expect nearer distances to cause an increase in the effect that LCA has on the accommodation response.

The results of the fitted linear mixed model are shown in Table 2. A model was also fitted that included experiment as a fixed effect and its interaction with distance and defocus; however no significant effect of experiment was found, and LRT and AIC model comparisons revealed that the model including experiment as a factor was not significantly better than the simpler model [ΔAIC = –45.85; χ^2^ (8) = 5.26; *p* = 0.730]. This means that the differences in the design of the three experiments did not influence the extent to which participants correct for LCA, when the individual differences among participants had been accounted for. Other fits including the median pupil diameter of participants as an interacting factor were also attempted; however, this variable was not found to have a significant effect.

**Table 2.** Linear mixed model results of accommodation to different wavelengths relative to the green illuminant, as a function of distance, the defocus caused by LCA, and their interaction. Coefficient estimates, their 95% CIs, and the random effects standard deviations (RE SDs) are shown, as well as the *t*-test results with degrees of freedom (*df*), *t*-ratios, and *p* values. Parameters in bold are significant at the 0.05 level. The fitted model says (Accommodation for this wavelength at this distance, relative to accommodation for 527.5 nm at this distance) = 0.05 − 0.02 × (Distance in diopters) − 0.26 × (LCA defocus in diopters) + 0.28 × (LCA defocus in diopters) × (Distance in diopters).

Parameter	Estimate	95% CI	*t*-ratio	*df*	*p*	RE SD
Intercept	0.05	−0.03 to 0.14	1.19	5205	0.233	0.19
Distance (D)	−0.02	−0.05 to 0.00	−1.71	5205	0.088	0.05
LCA defocus (D)	−0.26	−0.76 to 0.23	−1.04	5205	0.296	1.14
Distance (D) × LCA defocus (D)	**0.28**	**0.08 to 0.47**	**2.82**	**5205**	**0.005**	**0.45**

As seen in Table 2, the results agree with the previous slope estimations, with the effect of LCA defocus on accommodation increasing by a factor of 0.28 for every diopter of increase in the distance of the stimulus [95% CI, 0.08–0.47; *t*(5205) = 2.82; *p* = 0.005]. This means that the slope becomes 1 at a distance of approximately 4.5 D (–0.26 + 0.28 × 4.5 = 1). That is, for objects at around 22 cm, participants change their accommodative responses to compensate for the defocus caused by LCA to the full extent predicted by the chromatic eye model (Thibos et al., 1992). Furthermore, we see that, at a distance of 0 D, participants do not significantly change their accommodation to different wavelengths, with an estimated –0.26, not significantly different from zero [*t*(5202) = 1.04; *p* = 0.296], albeit there is considerable variability among subjects at this distance, as indicated by the large standard deviation of the random effects and the wide confidence intervals. Finally, as the predictor was centered at 527.5 nm and the response was centered by the 527 nm and 528 nm illuminants, effectively removing the effect of distance, we see that distance had no significant effect on accommodation when LCA defocus was 0 (i.e., at 527.5 nm).

**Figure 10.**
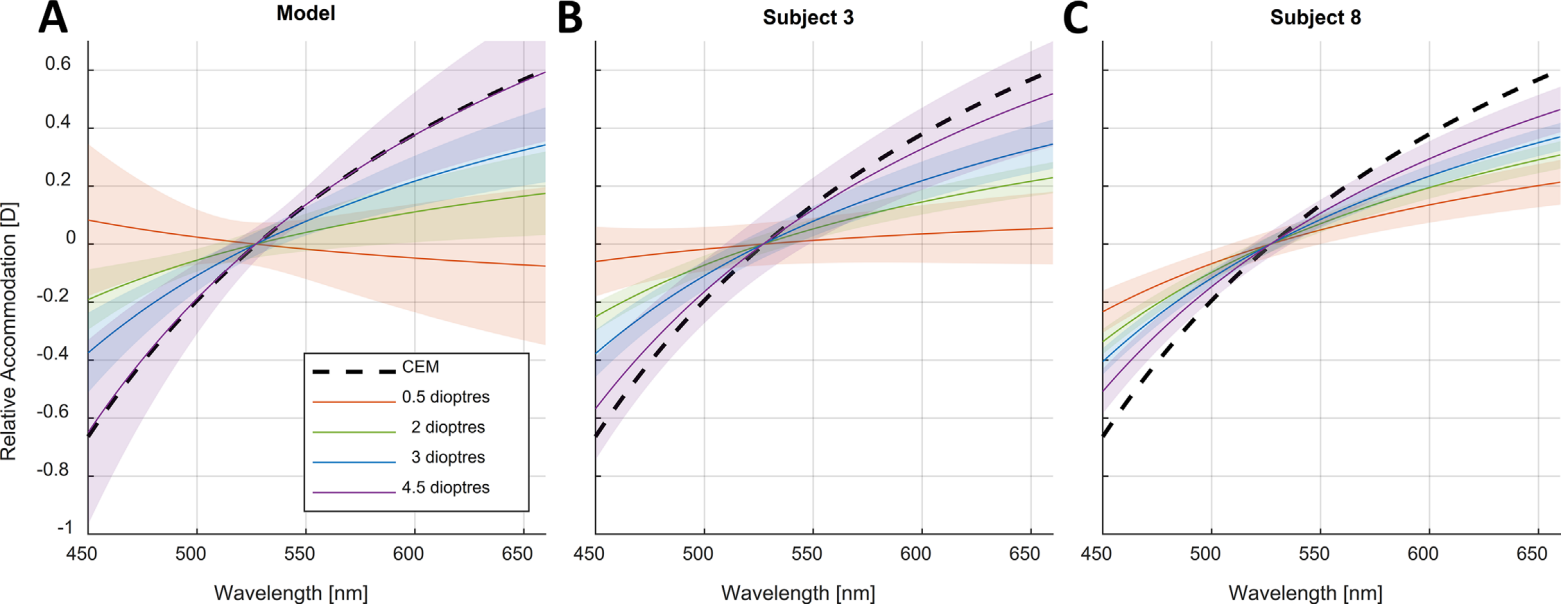
Relative changes in accommodation to different wavelengths as predicted by the linear mixed model (**A**) and for subjects 3 and 8 (**B** and **C**, respectively). The response at each distance was centered by the 527-nm and 528-nm illuminants, so it represents the relative difference in accommodation to these wavelengths. The black dashed line represents the defocus caused by LCA as predicted by the chromatic eye model (and centered at 527.5 nm). The continuous colored lines represent different distances as indicated by the legend, and the shaded regions represent the corresponding 95% CIs. This figure can be generated with the fig_i_LCAwithDistance.m file in the code repository, and it uses the shadedErrorBar function by Campbell (2023).

An illustration of the results of the model plotted as a function of wavelength and at different distances is shown in Figure 10A, as well as the fitted responses of two example subjects (Figures 10B and 10C). As observed, the confidence intervals are wider at the distance of 0.5 D, reflecting the uncertainty of the predictions likely caused by the interobserver differences being greater at this distance. This is illustrated in the differences between subject 3 and subject 8, as the latter shifted their accommodative responses to correct for LCA to a greater extent than the former when the stimulus was placed at 0.5 D. However, we see that for nearer distances, their responses are more similar. In summary, distance had a significant effect on the dioptric shift observed in the accommodative responses of participants to narrowband illuminants of different wavelengths; however, there was considerable intersubject variability, particularly at farther distances.

### Variability in the accommodation responses to narrowband and broadband illuminants

In our experiments, we recorded the refraction of the eye dynamically at a frequency of 50 Hz, which allows us to assess the within-trial variability of the steady-state accommodation response over time for the different illuminants used. In other words, when participants accommodate to a target, how much does the response fluctuate over time and are there any differences between narrowband and broadband illuminants and between narrowband illuminants of different wavelengths? Furthermore, as microfluctuations in accommodation are known to increase with increasing accommodative power, we also evaluated the effect of the mean refractive state as a predictor.

**Figure 11.**
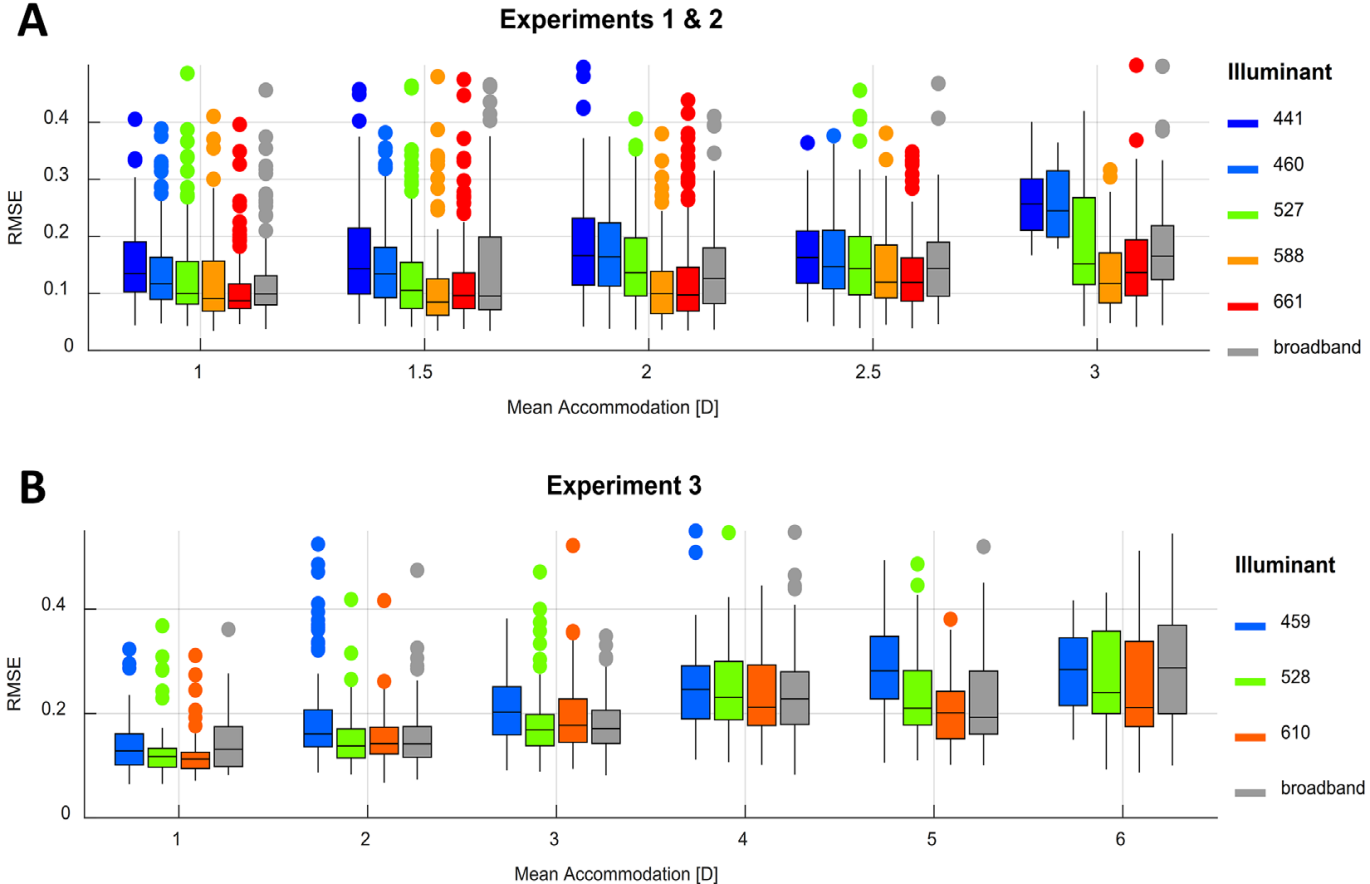
Distributions of the RMSEs of an unconstrained linear fit through the within-trial accommodation response, as a function of mean accommodation and illuminant in Experiments 1 and 2 (**A**) and in Experiment 3 (**B**). Each color represents an illuminant as indicated by the legend. The mean accommodation values have been rounded and grouped for illustration purposes only. Note the wider range of the horizontal axis for Experiment 3. This figure can be generated using fig_h_RMSEplot.m in the code repository, and it uses the Gramm toolbox by Morel (2018).

To obtain a measurement of intratrial accommodation variability, we fitted a linear function through the refractive response measured in each trial as a function of time and obtained the root-mean-squared errors (RMSEs). This approach has been used previously for similar purposes (MacKenzie, Hoffman, & Watt, 2010) and has the advantage of penalizing larger fluctuations in accommodation more and maintaining the units of the response. In Figure 11, we illustrate the distributions of the RMSEs as a function of mean accommodation and illuminant. Because the same illuminants were used in Experiments 1 and 2, the data obtained in both were combined. As shown, the within-trial variability seems to increase with increasing mean accommodative state, as well as appearing to be higher for shorter wavelength illuminants than longer wavelength ones, with no obvious differences observed between the latter and the broadband illuminants.

To quantify these differences, we fitted two linear mixed-effects models on the logarithmically transformed RMSEs, with mean accommodation as a continuous predictor and illuminant as a categorical predictor, while maintaining the full random-effects structure. The results are shown in Supplementary Table S4.

In both experiments, we observed similar intercepts of 0.14 D (95% CI, 0.11–0.17) in Experiments 1 and 2 and 0.13 D (95% CI, 0.11–0.16) in Experiment 3, for the 441-nm and the 459-nm illuminants, respectively. This means that at 0 D of refractive power, the accommodation response of observers to targets illuminated by these short-wavelength illuminants fluctuates on average by 0.13 and 0.14 D around the central response over time. The effect of accommodation on RMSE was similar in both experiments, as well, with 1 D of increase in accommodation causing an increase of 14.95% in variability (95% CI, 8.6–21.7) in Experiments 1 and 2 and an increase of 19.13% in variability (95% CI, 10.1–28.9) in Experiment 3. This agrees with several previous findings that the amplitude of accommodative microfluctuations increase with accommodation (Charman & Heron, 2015).

When comparing the different illuminants, we again saw similar results in both datasets, with the highest within-trial variability in accommodation being observed for the shortest wavelength illuminants, and this variability decreased as the peak wavelength of the illuminant increased.

In summary, we see that the within-trial variability of the accommodation response increases with increasing accommodation. It is lowest for the longer wavelength illuminants (588, 610, and 661 nm) and highest for the shorter wavelength illuminants (441, 459, and 460 nm). We found no systematic differences between broadband and narrowband illuminants, with the intratrial variability being similar for the middle-wavelength (527 and 528 nm) and broadband illuminants.

## Accommodation and pupil size

**Figure 12.**
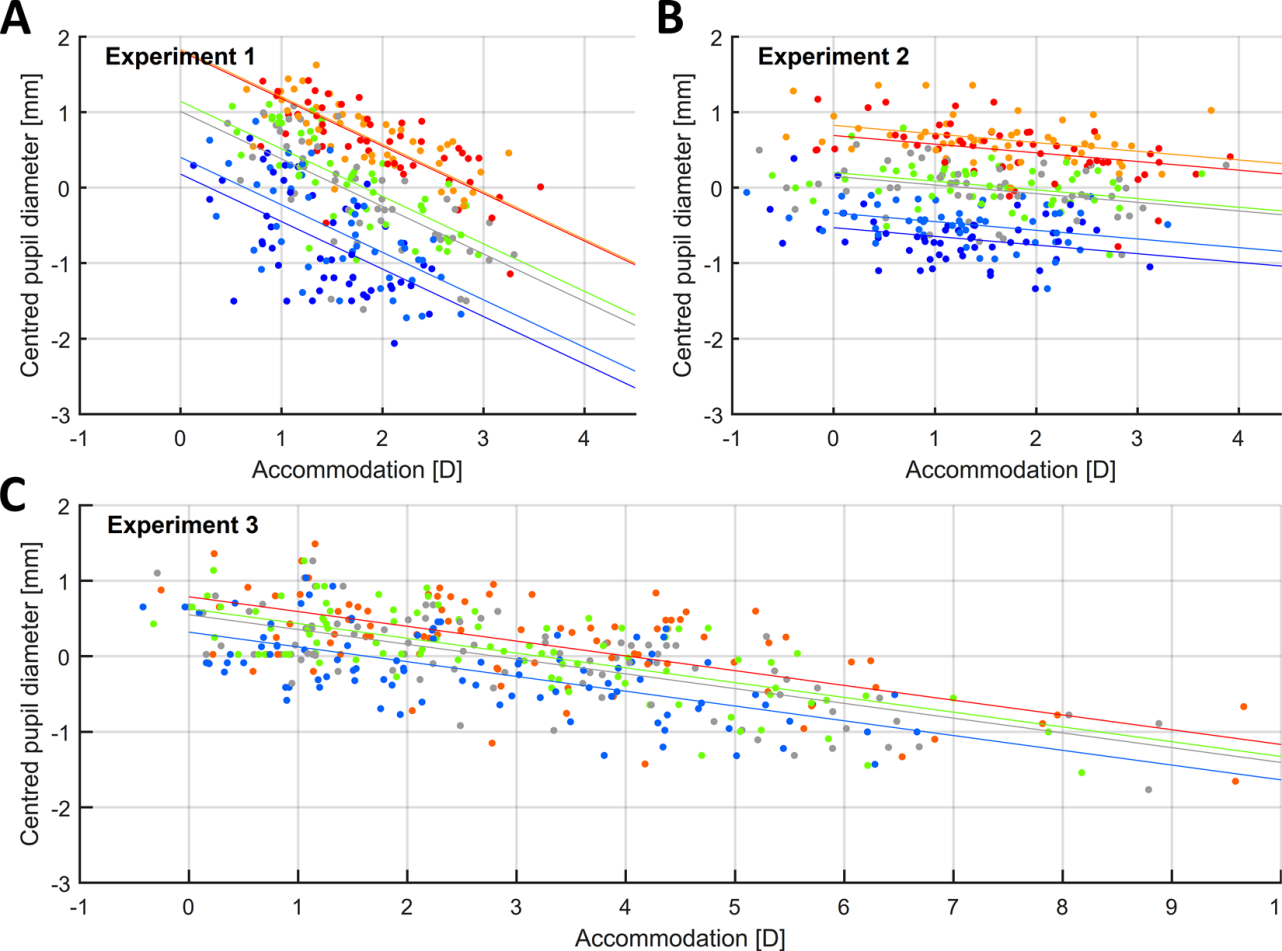
The median pupil diameter, centered to each participant, as a function of accommodation. For centering, we first subtracted the mean pupil diameter, averaged over all trials recorded for that participant. We have plotted the median of this centered value, averaged over all trials for a given illuminant and participant. Each panel represents the data obtained in one experiment, and the color of the points represents the illuminant used. As illustrated, pupil diameter decreased more steeply with increasing accommodation in Experiment 1 (where angular size was not kept constant) than in Experiments 2 and 3. Regression lines show, for illustration, fits to centered pupil diameter with fixed effects of illuminant and accommodation only. In practice, these are very similar to the mixed-effect model given in Supplementary Table S5. Note the wider range of the horizontal axis for Experiment 3. This figure can be generated with fig_e_PupilAcc.m in the code repository.

The median pupil diameter, centered to each participant, is illustrated in Figure 12 as a function of accommodation and for the different illuminants used. To assess the effect of accommodation and of the different illuminants on the pupil diameter of participants, we fitted a linear mixed model for each experiment, with accommodation in diopters as a continuous predictor and the illuminant as a categorical predictor, with random slopes and intercepts of participant. The latter were important, as there was significant interindividual variability in the median pupil diameter. The results are shown in Supplementary Table S5.

We found that pupil diameter significantly decreased as accommodation increased, although the rate of this change differed among the experiments. In Experiment 1, where the angular size of the stimuli increased as it was placed nearer the eye, pupil diameter decreased by 0.75 mm (95% CI, 62–89 mm) for every diopter of increase in accommodation. However, in Experiments 2 and 3, where the angular size of the stimuli was kept constant, the slope was shallower, with pupil diameter decreasing by 0.16 mm (95% CI, 0.06–0.26 mm) and 0.18 mm (95% CI, 0.06–0.29 mm) for every 1 D of increase in accommodation, respectively.

The different illuminants used had a significant effect on pupil size, with the shortest wavelength illuminants corresponding to the smallest diameters and pupil size increasing progressively for longer wavelengths, even though the luminance was equal in all cases. The largest differences in pupil diameter for stimuli of equal luminance were 1.70 mm (95% CI, 1.38–2.01) for Experiment 1 between the 441-nm and 661-nm illuminants, 1.40 mm (95% CI, 1.21–1.58) for Experiment 2 between 441 nm and 588 nm, and 0.45 mm (95% CI, 0.30–0.59) for Experiment 3 between 459 nm and 610 nm. Thus, a change in the peak wavelength of the illuminant used can have a larger effect on pupil diameter than changes in accommodation, particularly when the angular size of the stimuli is kept constant. Finally, the median pupil size for the broadband stimuli used seems to approximately correspond to the pupil diameter of middle wavelength illuminants: 527 nm in Experiments 1 and 2 and 528 nm in Experiment 3.

### Accommodative error and visual acuity

**Figure 13.**
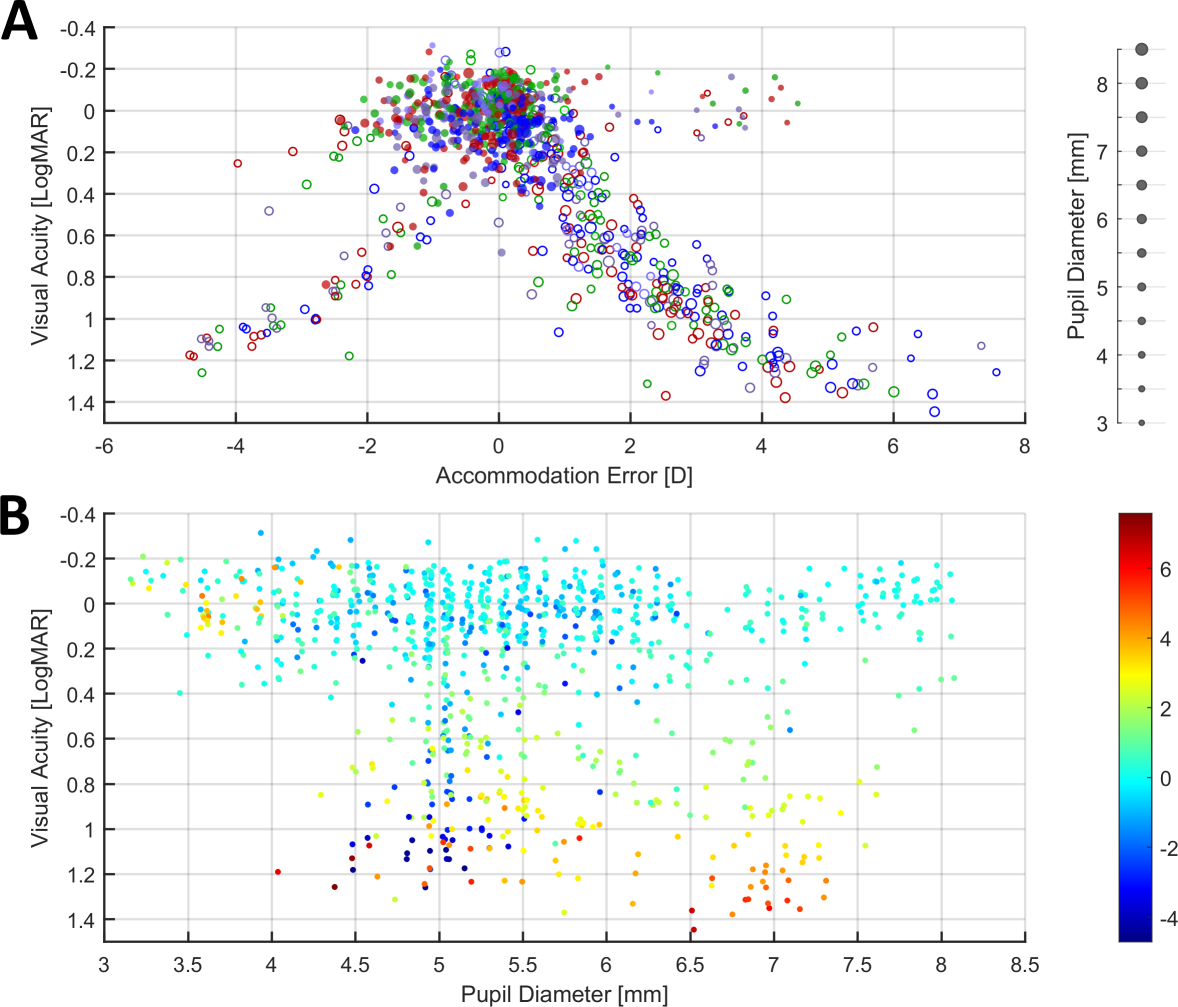
Visual acuity as a function of median accommodative error (**A**) and as a function of median pupil diameter (**B**). Because lower values correspond to better acuity, note that the *y*-axes are inverted. (**A**) Filled markers correspond to measurements over the linear portion of the accommodation response curve, and open markers correspond to measurements at distances where the function was saturated. The marker colors represent the illuminant used, and the marker sizes represent the corresponding median pupil size. (**B**) The color of the markers represents the median accommodative error. This figure can be generated with fig_g_VA_accError.m in the code repository.

In Experiment 3, the visual acuity of participants was measured for each illuminant at each distance, which allowed us to assess the effect that the median accommodation response of participants while they performed the staircase procedure had on their visual acuity. In Figure 13 we present the visual acuity thresholds obtained for all participants as a function of the median accommodative error (Figure 13A) and as a function of the median pupil diameter (Figure 13B). Individual figures for each participant are presented in Supplementary Figure S5.

First, we analyzed the results for stimuli that were within the subject's accommodative range, defined above as being the linear portion of the accommodation response curve (see Figure 7). These are shown with filled markers in Figure 13A. As observed, over this portion, participants had visual acuity thresholds that were mostly concentrated between –0.2 and 0.2 logMAR, which corresponds with better than normal to near normal vision. Median accommodative errors were mostly between –2 D and 1 D, with errors of larger magnitude mostly present where the accommodative demand was out of the subject's accommodative range (empty symbols) or when the pupil was small (small dots). The largest positive errors were of course obtained when demand was negative; that is, the participant was viewing the screen at 1 meter through a +2-D lens. When looking at the individual results of each participant (Supplementary Figure S5), we see that most of the data points with large negative errors of up to –2 D and low visual acuity thresholds belong to subjects 16 and 19, which were the two participants who presented the typical lags in their accommodation response curves. Thus, it seems that, in these two subjects, such lags did not correlate with a worsening of visual acuity.

Another relevant feature of the data is the small cluster of trials in which participants obtained low visual acuity thresholds between –0.2 and 0.2 logMAR, despite presenting positive accommodative errors of up to 4.5 D of magnitude. As illustrated in Figure 13B, one common feature of these trials is that the median pupil diameter of participants was mostly between 3 and 4 mm. Smaller pupil sizes improve depth of focus, which can decrease the effect that accommodative errors have on visual acuity. Additionally, the infrared photorefractor used relies on measuring the variation in reflected light intensity across the pupil to estimate the refractive state of the eye. This means that smaller pupils offer less information, which could lead to less accuracy in the measurements taken. For these reasons, and because at these small pupil sizes the measured accommodative error does not seem to correlate with visual acuity thresholds, data points where the median pupil diameter was below 4 mm were excluded from all analyses.

To further explore the relationship between accommodative error and visual acuity over the linear portion of the accommodation response curve, we fitted a linear mixed model with predictors of accommodative error magnitude, error sign, and their interaction, as well as illuminant and random intercepts and slopes of participants. The data used were the accommodative errors and visual acuity thresholds obtained within the accommodative range of participants (see filled markers in Figure 13A); trials were excluded where the median pupil diameter was smaller than 4 mm. The estimated coefficients are shown in Supplementary Table S6. We found that accommodative error magnitude was estimated to have a worsening effect on visual acuity, albeit the confidence intervals were wide, and the effect was not found to be significantly different from zero. The wide CIs likely reflect the fact that the errors over the linear portion of the accommodation response curve were very small in magnitude for most subjects. In other words, subjects were accommodating successfully to the stimuli over a range of distances, resulting in small values of defocus and greater uncertainty in estimating its effect on visual acuity. However, the parameter estimates still indicate that the overall effect on visual acuity was detrimental, with thresholds worsening by 0.10 logMAR for each diopter of increase in negative accommodative error [95% CI, –0.05 to 0.24; *t*(8.37) = 1.51; *p* = 0.168], and by 0.12 logMAR for each diopter of increase in positive accommodative error [95% CI, 0.00–0.23; *t*(6.44) = 2.36; *p* = 0.053].

A significant effect of illuminant on visual acuity was found. When accommodative error is zero, visual acuity for the 459-nm illuminant was estimated to be 0.04 logMAR (95% CI, –0.06 to 0.15). In comparison with this illuminant, visual acuity thresholds were lower for the 528-nm illuminant by 0.11 logMAR [95% CI, 0.06–0.17; *t*(8.7) = 3.95; *p* = 0.004], for the 610-nm illuminant by 0.08 logMAR [95% CI, 0.02–0.14, *t*(8.7) = 2.72, *p* = 0.024], and for the broadband illuminant by 0.09 logMAR [95% CI, 0.05–0.12; *t*(9.1) = 4.53; *p* = 0.001]. Post hoc pairwise comparisons of the estimated marginal means of visual acuity for each illuminant (i.e., the means averaged over the effects of accommodative error magnitude and sign) revealed that these differences were consistent and present across the small values of accommodative error found within the linear portion of the accommodation response curve. We found higher visual acuity thresholds for the 459-nm illuminant when compared to the 528-nm illuminant by 0.11 logMAR [95% CI, 0.02–0.20; *t*(8.91) = 3.87; *p* = 0.017], and by 0.09 LogMAR when compared to the broadband illuminant [95% CI, 0.02–0.15; *t*(8.74) = 4.34; *p* = 0.009]. Visual acuity was also lower for the 610-nm illuminant when compared to the 459-nm one by 0.08 logMAR, although this difference was not significant [95% CI, –0.01 to 0.18; *t*(8.91) = 2.66; *p* = 0.100]. This means that, over the linear portion of the accommodation response curve and for equal values of accommodative error, visual acuity was worst for the shortest wavelength illuminant than for any of the other illuminants used. No significant differences were found in pairwise comparisons between the 610-nm, 528-nm, and broadband illuminants. In other words, at least in this low luminance regime, acuity is the same for stimuli presented on the green and red primaries but is one line worse on the Snellen chart for stimuli on the blue primary.

To further explore the effect of accommodative error and the illuminants used we fitted linear mixed models on all of the data obtained, including distances that were nearer or farther away than the participant's accommodative range (see Figure 13B, both open and filled markers). Due to the complexity of the data and the observed differences between the effect of underaccommodation (negative errors) and overaccommodation (positive errors), the dataset was separated accordingly and fitted separately. For positive accommodative errors, visual acuity thresholds seem to saturate for error magnitudes greater than 5.5 D and at around 1.2 logMAR; thus, these values (error magnitude > 5.5 D and visual acuity > 1.2 logMAR) were excluded from the analyses to improve model convergence. As with the previous model, trials where the median pupil diameter was less than 4 mm were excluded, and the pupil diameter predictor was centered so that the intercept of the model was at 4 mm. Several models were fitted to both datasets, with different combinations of accommodative error magnitude, pupil diameter, illuminant, and retinal illuminance used as separate or interacting predictors while maintaining the full structure of the random effects. Through multiple comparisons, it was determined that, for both datasets, a model with predictors of error magnitude, pupil diameter, their interaction, and illuminant had the greatest predictive power and lowest AIC. The results of the fits for both datasets are shown in Supplementary Table S7.

For overaccommodation (Supplementary Table S7, top), we see that the accommodative error magnitude had a significant effect on visual acuity, with thresholds worsening by 0.21 logMAR for every 1-D increase in error for a pupil diameter of 4 mm [95% CI, 0.10–0.31; *t*(4.38) = 3.84; *p* = 0.016]. Furthermore, for every millimeter of pupil size increase, the effect of error magnitude on visual acuity significantly increased by 0.08 logMAR [95% CI, 0.02–0.13; *t*(5.39) = 2.78; *p* = 0.036]. This means that, when participants have larger pupil sizes, their visual acuity is more affected as defocus increases. No significant differences in visual acuity were found between illuminants, so the differences previously observed for small accommodative errors within the linear portion of the accommodation response curve are not present for positive accommodation errors of larger magnitude.

For underaccommodation (Supplementary Table S7, bottom), we see that increases in error magnitude had a smaller effect on visual acuity that did not reach significance, with thresholds only worsening by 0.06 logMAR [95% CI, 0.00–0.12; *t*(9.97) = 1.82; *p* = 0.098] for every diopter of increase in error and a pupil diameter of 4 mm. For each 1 mm of increase in pupil diameter, the effect of accommodative error increased by 0.04 logMAR per diopter; however, the CIs are wide, and the effect is not significant [95% CI, –0.02 to 0.11; *t*(3.53) = 1.31; *p* = 0.268]. Finally, visual acuity thresholds were higher (i.e., acuity was worse) for the 459-nm illuminant when compared to the 528-nm illuminant by 0.07 logMAR [95% CI, 0.01–0.14; *t*(7.31) = 2.16; *p*=0.066] and the broadband illuminant by 0.07 (95% CI, 0.01–0.14; *t*(4.38) = 2.15; *p* = 0.086]; however, these differences only reach significance at the 0.10 level.

The differences in results between both models could be explained by the fact that the negative accommodative errors were found mainly over the linear portion of the accommodation response curve, as the nearest distance used was not sufficient to reach the upper limit of the accommodative range of most participants. Indeed, we can see the similarities between the results for the linear portion of the accommodation response curve and for all of the negative accommodative errors data. On the contrary, most participants did reach the lower limit of their accommodative range before the farthest distances used, so there was a wider range of data for the positive accommodative errors fit. However, it is possible that some of the differing results found were due to inherent differences in the effect of the accommodative error sign, as we see that for one of the two participants who reached their upper accommodative limit visual acuity thresholds increased with a shallower slope when underaccommodating to the stimuli (see Supplementary Figure S5, subject 2).

## Discussion

In this study, we performed three experiments where we measured the steady-state accommodation and pupil responses of mostly untrained observers when looking at targets illuminated by different narrowband lights and placed at different distances.

### Effect of stimulus spectrum on variability of accommodation

We found that most participants were able to accommodate under monochromatic light when the illumination of the target was changed abruptly and were able to maintain focus for the duration of the trials with similar variability as in white light, particularly at nearer distances. Like earlier workers (Charman & Heron, 2015), we found that the within-trial variability of the accommodation response increased with accommodation. We also found that variability increased slightly for shorter wavelengths at a given distance, perhaps reflecting the greater LCA (cf. longer vertical error-bars in Figure 3C). However, we found no systematic differences between broadband and narrowband illuminants in the variability of the accommodation response of observers over time, and the within-trial accommodative response fluctuated on average by similar amounts for the broadband and the green illuminants.

This finding contradicts some of the results reported by Kruger et al. (1997), as 38% of their sample had difficulty maintaining focus with narrowband targets placed away from the tonic state accommodation (at distances of 0 and 5 D), but they could accommodate accurately to a broadband target at the same distance. These distances were included in our experiments, and we found no such impairment in steady-state accommodation. Instead, our results agree with those of Atchison et al. (2004) that accommodative responses to targets with reduced spectral bandwidth were not more variable than responses to broadband targets, as well as with the finding of Charman and Tucker (1978) that participants can accommodate to narrowband stimuli of different wavelengths and maintain focus as accurately as in white light. It is notable that most of the participants in our sample were untrained naïve observers, as the one inexperienced observer of Charman and Tucker (1978) was not able to accommodate to the narrowband stimuli without additional training in the task. It is plausible that today, with the increasing prevalence of narrowband LEDs as primaries in digital displays and as illumination sources, naïve observers have more experience accommodating to this type of stimulus and can make use of other cues to determine the sign and magnitude of the accommodative change, as well as to maintain focus. Some residual chromatic blur could still be present in our narrowband stimuli that could potentially serve as an accommodative cue, as LEDs are not completely monochromatic (with a spectral bandwidth of ∼20 nm). This would be especially true for shorter wavelengths, as the effects of LCA are greater toward the blue end of the spectrum. However, we did not find that accommodation was more accurate for short wavelengths, and, in fact, the slope of the accommodation response curve as a function of distance was shallowest for these illuminants and the variability of the response was higher. Thus, there is no evidence that any residual chromatic blur within a single narrowband illuminant contributed to the subjects’ abilities to accommodate.

### Accommodation compensates for defocus due to LCA better at near distances than far

One of our main findings is that the slope of the linear portion of the accommodation stimulus–response curve becomes shallower as the peak wavelength of the narrowband illuminants decreases, which is caused by an increase in the difference in accommodation to different wavelengths as the target is placed at nearer distances. In other words, the extent to which participants change their accommodation responses to compensate for the LCA of the eye increases as they accommodate to nearer targets. At a distance of ∼0.5 D, there are no significant differences in the accommodation to different wavelengths, but, at approximately 4.5 D, participants change their accommodation responses nearly to the full extent that the chromatic eye model predicts (Thibos et al., 1992). This was a common finding in all three experiments, but there were considerable differences between participants, particularly at farther distances, as some subjects did change their accommodative responses to some extent to compensate for LCA even at 0.5 D or farther.

Importantly, this does not just trivially reflect the nonlinear mapping from distance to diopters. A range of ±0.1 D centered on 0.5 D (2 m) corresponds to a distance of 83 cm, and the same range centered on 4.5 D (22 cm) corresponds to a distance of just 1 cm. Thus, it is natural for accommodation to be more precise in meters at near distances. However, as regards LCA, it is also more precise in diopters. As a concrete example, the chromatic eye model predicts that around 1.2 D more accommodation is needed to focus on red stimuli at 650 nm than on blue at 450 nm. For nearby stimuli at 4.5 D, our subjects do indeed on average show around 1.2 D more accommodation for red than blue. However, for distant stimuli at 0.5 D, most show no significant difference in accommodation for red vs blue stimuli. Thus, for narrowband stimuli, accommodative error in diopters is larger on average for distant stimuli, as shown in Figure 9.

This might partly be because observers reach the far point of their accommodative range at nearer physical distances for targets illuminated by shorter wavelength light. In other words, the typical lag of the accommodation response curve would start to occur at nearer distances for short- than for long-wavelength light, which could cause the slope estimate to be shallower. However, we were careful to fit slopes only to the linear portion of the accommodative response for each illuminant individually, before this saturation would apply. Thus, we do not think this can entirely account for our results.

Charman and Tucker (1978) found some comparable results. They measured the accommodation response curves for six participants under red and blue light and found that the slope was shallower for blue (468 nm) in at least two of the subjects. However, for one of these subjects they measured the response to other narrowband illuminants (644 nm, 579 nm, 546 nm, and 503 nm) and did not find a significant difference in the slope of the accommodation function other than for blue, albeit at distances of 1 D and 2 D this subject significantly underaccommodated for red (644 nm). They theorized that this change could be partly explained by an increase in the LCA of the eye as the power of the crystalline lens increases. They then took objective measurements of the LCA of the eye in this participant and observed that it increased by ∼3% (0.03) per diopter of accommodation, which they postulated could account for the results found for that subject (although some overaccommodation for blue and underaccommodation for red remained at the farthest distances tested of 1 D and 2 D, even after this adjustment). Across our sample, however, we found that the extent to which participants changed their accommodation responses to correct for LCA increased by a much larger factor of 0.28 (95% CI, 0.08–0.45) per diopter of increase in accommodative demand; thus, although an increase in LCA with accommodation might account for part of our results, it does not seem to fully explain them on its own. Charman and Tucker (1977) proposed that the change in slope in blue light might be due to reduced acuity at shorter wavelengths; however, we found that the difference in slope was significant between other illuminants tested, as well (e.g., red and orange), so it does not seem to be unique to blue light.

Previous studies have found an increase in LCA with accommodation (Nutting & Larmor, 1997; Sivak & Millodot, 1974), as well as interindividual differences in the LCA measured for different observers (Bedford & Wyszecki, 1957; Nutting & Larmor, 1997; Sivak & Millodot, 1974; Wald & Griffin, 1947). Sivak and Millodot (1974), in particular, used an achromatizing lens that corrected for most of the LCA of the eye (Bedford & Wyszecki, 1957) and subjectively measured the difference in optimal focal distance between different wavelengths at distances of 0.6, 3.0, and 7.1 D. They observed that the difference in accommodation as a function of wavelength increased in all subjects from a mean of 0.40 D at the farthest distance to 0.65 D at the nearest, with some variability among observers. If we perform a linear fit on their data, we see that the rate of increase in LCA is 0.036 (or 3.6%) per diopter of accommodation, similar to the results of Charman and Tucker (1977).

The fact that participants accommodate with increased accuracy to different wavelengths as the target nears is perhaps a surprising result, if we consider our finding that pupil size decreases with increasing accommodation, increasing the depth of focus of the eye. Indeed, it has been observed that the steady-state accommodative response of the eye is more accurate for larger pupil sizes (Ward & Charman, 1985); thus, we would expect observers to compensate for LCA to a greater extent when pupil size is larger at farther distances. In addition to this, in the first experiment the angular size of the target increased as it was placed nearer the observer, which would have decreased the high spatial frequency content of the image and increased power at lower spatial frequencies. Previous studies have found that the steady-state accommodation response is more accurate for higher spatial frequencies and substantial in error for lower spatial frequencies (Charman & Tucker, 1977), so we would expect this factor to contribute to responses being less accurate at nearer distances, but the results of this experiment indicate otherwise.

One possibility that could explain these results is that, as LCA is significantly reduced in narrowband light, participants are making use of other cues to find the optimal focal distance for different wavelengths, and these cues might change with distance. Specifically, the microfluctuations of the crystalline lens have been found to increase in magnitude as accommodation increases due to the increased freedom of movement (Day, Strang, Gray, & Seidel, 2006; Kotulak & Schor, 1986; Stark & Atchison, 1997), covering an approximate range of 0.02 D in both directions when the mean accommodation is 1 D and increasing to a range of up to 0.1 D when the accommodative response is 4 D. These microfluctuations could serve as a cue to accommodation by providing negative feedback to the accommodative control mechanism, essentially functioning as a subthreshold blur detector; thus, it is possible that the increased range of these microfluctuations at higher accommodation levels allows the visual system to find the focal distance for each wavelength more accurately when the color of the target is changed and in the absence of the chromatic blur caused by LCA. However, this is only speculation on our part, as there is no evidence in the literature that the increased amplitude of microfluctuations can lead to higher accommodative accuracy; on the contrary, consistent steady-state errors when accommodating to nearer targets are often found (Plainis, Ginis, & Pallikaris, 2005).

Overall, our results seem novel within the literature, although Charman and Tucker (1978) had some comparable findings with two of their subjects. It is not clear why participants increasingly correct for LCA at nearer distances when accommodating to narrowband stimuli, and more research is needed in this area to explain these results, as well as to further explore the individual differences among observers.

### Accommodation to broadband white light

Another of our findings was that accommodation to white light tended to overlap with middle wavelengths over all distances tested (see Figures 6 to 9). When the targets were illuminated by a white light with the highest luminous spectral power between 530 and 590 nm, accommodation was similar to the narrowband illuminants of similar peak wavelengths (527 and 588 nm) over all distances tested, although the slope as a function of distance was steeper than for the narrowband illuminants and closer to 1, with accommodation slightly shifting from green toward orange as accommodative demand increased from 0.5 to 3 D. In a third experiment, where a broadband illuminant was created by using the three narrowband primaries at equal luminance, accommodation seemed to overlap with the red illuminant (610 nm) or between the red and green (528 nm) illuminants over most distances tested.

Some previous studies have investigated the wavelength that comes into focus in the retina in broadband white light at different distances. Ivanoff (1949) found that, with increasing accommodation, the wavelength that was kept in focus in the retina decreased, from ∼600 nm at 0.5 D to ∼500 nm at 2.5 D. Similarly, Sivak and Millodot (1974) found that the wavelength in focus changed from 620 nm at a distance of 0.7 D to 530 nm at a distance of 7.1 D. Ivanoff (1949) proposed that this change of wavelength in focus with distance could explain the lag and leads of the accommodative response by a process of “sparing of accommodation”; that is, the visual system uses the LCA of the eye to accommodate as close as possible to the tonic or resting state, choosing to accommodate to shorter wavelengths at near distances, as they require the least refractive power, and to longer wavelengths for farther distances. If this were the case, one would expect to find much steeper stimulus–response curves for narrowband illuminants than for white light, which does not agree with our findings. Similarly, Charman and Tucker (1978) and Jaskulski et al. (2016) did not find that the stimulus–response curves for narrowband light of different wavelengths were steeper than for white light. Thus, no “sparing of accommodation” seems to be taking place, and it is possible that those earlier findings were due to the spherical aberration of the eye usually changing from positive at far to increasing negative values as accommodation increases (Del Águila-Carrasco, Kruger, Lara, & López-Gil, 2020; Thibos, Bradley, & López-Gil, 2013). When spherical aberration is positive at far distances, the rays entering through the periphery of the pupil will come into focus in front of the retina, so the shorter wavelength content of that light will be more out of focus and longer wavelengths will come into focus closer to the retina. When accommodation increases and spherical aberration becomes negative, the peripheral rays will come into focus behind the retina, so the longer wavelength content will be more out of focus and shorter wavelengths more in focus. Thus, it is possible that the phenomenon observed by Ivanoff (1949) was due to the distribution of light in the retina changing due to a change in the sign of the spherical aberration of the eye, rather than by the visual system shifting the wavelength that is kept in focus in white light.

Our results seem to indicate that, when accommodating to white light, the wavelength that is kept in focus is between 527 and 610 nm, which agrees with findings by DeHoog and Schwiegerling (2007) that the best focus in white light corresponded to best focus for monochromatic light between 590 and 610 nm. The slightly steeper slope, closer to unity, that we found for white light when compared to the green or orange illuminants could be because chromatic blur due to LCA aids accommodation.

### Effect of accommodation and wavelength on pupil size

Another of our findings was that steady-state median pupil diameter decreased with increasing accommodative state and with decreasing peak wavelength in the narrowband illuminants, even when luminance and angular size were equal (see Figure 12). The effect of wavelength on narrowband illuminants can be explained by the contribution of the melanopsin photopigment present in some retinal ganglion cells (intrinsically photosensitive RGCs [ipRGCs]) to steady-state pupil size control (Spitschan, 2019a; Spitschan, 2019b). Although our stimuli were created to provide equal input to the luminance channel, pupil size control has a strong input from the ipRGCs in addition to the cone photoreceptors. The melanopsin photopigment is more sensitive to short-wavelength light than the L- and M-cones, with a peak sensitivity at 480 nm (Enezi et al., 2011); thus, the shorter wavelength light used in our experiments would provide greater stimulation to the ipRGCs and the pupil control mechanism than the longer wavelength illuminants of equal luminance.

The literature investigating the effect of accommodation on pupil size offers a less clear picture to explain our results. Although the near triad of accommodation, convergence, and pupil constriction is a well-established fact, there is contradictory evidence on whether convergence or accommodation are responsible for the pupil response at near distances. Some studies have found that accommodation alone does not trigger a pupil response when convergence and other factors such as target size and alignment are controlled (Feil, Moser, & Abegg, 2017; Phillips, Winn, & Gilmartin, 1992; Stakenburg, 1991). However, one of these studies measured dynamic rather than steady-state pupil responses, and another did not directly measure accommodation but inferred it from acuity measurements. Other studies have arrived at the opposite conclusion, finding that blur-driven accommodation and not fusional vergence caused pupil constriction (Marg & Morgan, 1949; Phillips et al., 1992; Wilson, 1973). As to the extent of the change, Marg and Morgan (1950) found that pupil diameter changed on average by 0.48 mm per diopter of accommodative stimulus and did not change with convergence, although it is possible that factors such awareness of target proximity might have played a role (Phillips et al., 1992). On the other hand, O'Neill and Stark (1968) and van der Wildt and Bouman (1971) both reported measurements showing an increase of ∼0.17 mm per diopter of accommodation when describing the design and construction of equipment to measure accommodation, vergence, and pupil diameter dynamically. Although their experiments had more carefully controlled parameters (presenting the targets monocularly and maintaining constant target size, alignment along the axis of the eye, and luminance), their samples were limited to just one subject each. Here, we present results with a larger sample that show similar estimates, with steady-state pupil diameter decreasing by 0.16 to 0.18 mm per diopter of accommodation for targets that were viewed monocularly, aligned with the axis of the stimulated eye, and with constant luminance and angular size. Furthermore, in one of our experiments the apparent distance of the target was also kept constant, with the accommodation being driven by placing lenses in front of the eye. Thus, our results provide further support to the idea that steady-state pupil constriction can be caused by accommodation alone, although the rate of change is smaller than reported in some of the previous studies.

### Effect of accommodative error and wavelength on visual acuity

Over the quasilinear portion of the accommodation response curve, we found that accommodative errors (i.e., the difference between accommodative demand and the median response) had mostly magnitudes of up to 1 D in either direction, although underaccommodation was more prevalent in our sample. An interesting finding was that not all subjects presented the consistent lags in accommodation as the target neared that are often reported in the literature (Nakatsuka, Hasebe, Nonaka, & Ohtsuki, 2003), and overall there was great intersubject variability in the shape and slope of the stimulus–response curve. Furthermore, the two subjects that did present significant lags of up to 1.5 and 2 D of magnitude at near distances in the third experiment did not have their visual acuity significantly impaired by those accommodative errors. In fact, over the linear portion of the accommodation response curve for all participants, we found that accommodative error did not have a significant effect on visual acuity thresholds.

It is possible that our measurements were not precise enough to capture the relationship between accommodative error and visual acuity for a relatively small range of errors. Accommodation was measured as participants performed the staircase procedure with targets of different spatial frequencies being presented and pupil size allowed to change freely, so the accommodative response would not be the only factor affecting retinal image quality, and the median of this response might not be representative of the defocus of the retinal image when participants were viewing the smaller targets that were more critical to the thresholds obtained. The depth of field of the eye would also allow some of these accommodative errors to not have a detrimental effect on visual acuity. For pupil diameters between 4 and 6 mm, the depth of field can be between 0.4 and 0.5 D, even for high spatial frequencies and monochromatic light (Marcos et al., 1999), albeit higher estimates have been obtained (Wang & Ciuffreda, 2006). Jaskulski et al. (2016), for example, found that the subjective depth of field for a pupil diameter of 3.8 mm and a target of 0.1 logMAR size, was approximately 1.19 D for narrowband light and slightly higher for polychromatic light. Of course, even the higher estimates are not enough to fully explain the results obtained, particularly in the two subjects that showed lags of significant magnitude.

Recently, Labhishetty et al. (2021) used several objective and subjective measurements to measure the accommodation of the eye. They found that, for target distances between 0 and 6 D, objective measurements had higher accommodative errors than subjective measurements based on visual acuity. In particular, the measurements taken using a photorefractor gave the largest measured lags, with magnitudes between 0.5 and 1.5 D. Despite these large errors, subjective measurements indicated that participants were accommodating accurately to the distance that maximized their visual acuity, with the subjective errors being much lower at ∼0.15 D. These results are comparable to ours, as we used a photorefractor to measure accommodation and found errors of considerable magnitude (mostly lags) that did not seem to have a detrimental effect on visual acuity. In particular, the two subjects who displayed the more typical large accommodative lags maintained visual acuity thresholds close to 0 logMAR regardless of the magnitude of these errors.

It has been suggested that the consistent errors that are observed when accommodation is measured objectively with a photorefractor (i.e., lags and leads) might actually be the consequence of the spherical aberration of the eye, particularly its change in sign with accommodation (Plainis et al., 2005; Thibos et al., 2013). As mentioned previously, the eyes of most observers tend to exhibit positive spherical aberration when accommodating at far distances, which decreases steadily with increasing demand and becomes negative at nearer distances (Del Águila-Carrasco et al., 2020). This means that peripheral rays will come into focus in front of the retina at far distances and behind the retina at near. As photorefractors use the distribution of reflected light across the entire pupil to estimate the refractive state of the eye, they might put more weight on these marginal rays than the visual system does, leading to apparent leads and lags in accommodation, even when paraxial rays are focused correctly in the retina (Thibos et al., 2013). Thus, it is possible that the large accommodative lags observed in two of the subjects in our third experiment are due to their own spherical aberration and the method used to measure accommodation.

Although accommodative errors were small over the linear portion of the accommodation response curve and thus did not have a significant effect on visual acuity, we did find differences caused by the illuminants used. Visual acuity was significantly poorer for blue light when compared with the other three illuminants. When accommodative error was zero, the visual acuity for blue light was estimated to be 0.04 logMAR; for the red, green, and broadband illuminants, the visual acuity values were –0.04, –0.07, and –0.05 logMAR, respectively. As luminance was kept the same for all illuminants, giving the same input to the luminance (L+M) channel, the differences in visual acuity cannot be explained by the differences in sensitivity to different wavelengths. The lower visual acuity for blue light can rather be explained by the blur caused by LCA for shorter wavelengths. For a spectral distribution that is not completely monochromatic, the LCA of the eye will cause greater defocus at shorter wavelengths, which will in turn reduce retinal image contrast, particularly at higher spatial frequencies. The blue light used had a spectral bandwidth (FWHM) of 20 nm around a peak wavelength of 459 nm, which would cause a difference in defocus of 0.21 D. In comparison, even though the green and red illuminants had slightly larger spectral bandwidths (25 and 28 nm, respectively), there would only be differences of 0.16 D and 0.11 D in defocus across the bandwidth of their respective spectral distributions. As this defocus is caused by the intrinsic change in refractive index of the eye with wavelength, the accommodative response alone cannot correct it. Furthermore, it seems that the smaller average pupil size for blue light is not sufficient to improve focus or the retinal contrast of the image. Indeed, it has been observed previously that, although visual acuity in blue narrowband light is lower under normal conditions, compensating for the LCA of the eye improves the thresholds so that they more closely match those obtained in green, red, and white light (Domenech, Seguí, Capilla, & Illueca, 1994). Overall, however, our results indicate that the visual acuity thresholds for blue were still within the range of normal vision, and the differences with the other primaries of the display were small (∼0.09 logMAR), such that it is unlikely to have a significant impact in most real-life applications.

The increased defocus caused by LCA for shorter wavelengths could also explain the increased variability of the accommodation response for these illuminants when compared with longer wavelength ones. It has been previously reported that the magnitude of the microfluctuations of accommodation increases with increasing blur in the image (Niwa & Tokoro, 1998) and with decreasing contrast (Charman & Heron, 2015) and correlates with the objective depth of focus of the eye (Yao, Lin, Huang, Chu, & Jiang, 2010). The higher defocus caused for narrowband LEDs of shorter peak wavelength would reduce retinal image contrast and increase the depth of focus, which could increase the magnitude of the microfluctuations of accommodation, resulting in the higher within-trial variability of the response observed. We did not find, however, an increased variability in the broadband illuminants used, even though defocus and depth of focus would be greater in this condition (Jaskulski et al., 2016). Niwa and Tokoro (1998) previously found that microfluctuations increase as the blur of the image increases, but only for small amounts of blur. As the magnitude of blur continues to increase, the magnitude of the microfluctuations begins to decrease again, and the amount of blur at which microfluctuations peak is lower for higher spatial frequencies. These changes were found in particular for the low-frequency components of microfluctuations which are caused by the action of the ciliary muscles on the crystalline lens (i.e., are under neural control) and may be of more significance to accommodative control (Charman & Heron, 2015). As blur increases, microfluctuations might increase in order to serve as an error signal to accommodation and improve the accuracy of the response. With higher depth of focus, the magnitude of the microfluctuations would have to be higher in order to provide the same amount of information for error detection to the accommodative control mechanism. (Jaskulski et al., 2016). Niwa and Tokoro (1998) postulated that, when blur surpasses a certain threshold, it can no longer be discriminated and there is an overall reduction in microfluctuations, with this threshold being lower for higher spatial frequencies as they are more affected by defocus. Thus, it is possible that the higher defocus caused by LCA for the broadband illuminants is not detectable, causing the magnitude of the microfluctuations to be lower when compared with the short-wavelength illuminants. It is important to note that these differences in response variability were found even after controlling for the mean accommodative state of the eye, as microfluctuations have been found to increase with increasing accommodation (Day et al., 2006; Kotulak & Schor, 1986; Stark & Atchison, 1997), which we corroborated in this study.

Interestingly, visual acuity thresholds in white light did not differ significantly from those obtained with the narrowband green and red illuminants. The chromatic blur caused by LCA in broadband white light did not seem to impair visual acuity, even though for our broadband illuminant all three primaries were set at equal luminance (so the chromatic blur would not be attenuated by the reduced luminous sensitivity at longer and shorter wavelengths). Domenech et al. (1994) found comparable results: With normal LCA, the visual acuity in white was similar to that for red and green narrowband light, and compensating for the LCA of the eye did not significantly improve the thresholds for white light. More recently, Suchkov, Fernández, and Artal (2019) also found that correcting the LCA of the eye did not cause the predicted improvement in visual acuity, but rather a slight decrease (albeit not statistically significant) and that doubling the LCA of the eye had a more detrimental effect than predicted from their simulations. Further evidence from these authors also shows that correcting for the LCA of the eye does not improve visual acuity in high-contrast conditions, even when subjects are given time to adapt to the corrected LCA (Fernandez, Suchkov, & Artal, 2020). Thus, it seems that the ability of the visual system to resolve small targets with precision is not impaired by the chromatic blur caused by LCA, at least in high-contrast conditions.

Finally, when considering all the visual acuity measurements, including those obtained beyond the accommodative range of participants, we found that overaccommodation had a significant detrimental effect on visual acuity and a statistically significant interaction with pupil diameter, such that visual acuity was more affected by defocus in larger pupils than in smaller ones. This is consistent with previous findings in the literature of an increased depth of focus with smaller pupil sizes (Marcos et al., 1999; Wang & Ciuffreda, 2006). No effect of illuminant was found, indicating that larger values of defocus affect broadband and narrowband targets equally, including blue light. This is also consistent with previous findings that indicate that depth of focus increases with decreasing acuity (Tucker & Charman, 1975), such that the small amount of blur caused by LCA for blue light would no longer have an impact on retinal image quality.

## Conclusions

In summary, we found that narrowband illumination can be an adequate stimulus to accommodation when compared with white light, even in a sample of mostly untrained observers. We also found that the extent to which participants change their accommodative responses to compensate for the LCA of the eye increases at nearer distances and matches the predictions of the chromatic eye model from approximately 4.5 D (22 cm) and nearer, a finding that is not fully explained by the previously reported increase in LCA of ∼3% per diopter of accommodation. This means that considering the spectral distribution of the display primaries and its effect on accommodation might be more relevant for displays that are used at nearer distances, such as mobile phones or computer monitors, than for those that are viewed farther away such as television or cinema screens. We found no detrimental effects on visual acuity for narrowband light, with only blue light causing a significant worsening of the thresholds due to the larger spread of defocus caused by LCA at shorter wavelengths for a display primary that is not completely monochromatic. However, visual acuity in blue light was still within the range of normal vision (∼0 logMAR), and this small difference is unlikely to be relevant to real-life display applications where images with multiple spatial frequencies are used.

## Supplementary Material

Supplement 1
