# Supplementary material for: Ocular accommodation and wavelength: The effect of longitudinal chromatic aberration on the stimulus–response curve
Source: J Vis. 2024 Feb 27;24(2):11. doi: 10.1167/jov.24.2.11 (PMC10910436; doi:10.1167/jov.24.2.11)
Supplement: Supplement 1 [file jovi-24-2-11_s001.pdf]

Supplement: Ocular accommodation and wavelength: the effect of longitudinal chromatic aberration on the stimulus-response curve.

Methods.

See main text for details. In brief, in experiments 1 and 2, the accommodation function was sampled by changing the physical distance of the stimuli, with the angular size of the diffuser changing concurrently in experiment 1 and being kept constant in experiment 2. In experiment 3, the accommodation function was measured by using trial lenses to simulate a larger range of optical distances, and the visual acuity of participants was measured concurrently.

Photorefractor calibration.

The photorefractor used in these experiments (PowerRef 3 from Plusoptix) consists of an array of nine infrared LEDs (850nm) located eccentrically below an infrared camera that records at 50Hz. Two mirrors reflect the infrared light into the retina (see Figure 2), which diffusely reflects this light back into the camera, and depending on the refractive state of the eye, the light reflected will vary in intensity vertically across the pupil. An inbuilt calibration factor then converts this slope of intensity across the pupil into a defocus value that indicates the refractive state of the eye.

This slope-based eccentric infrared (IR) photorefraction offers a convenient non-invasive way to measure refraction dynamically over a large range of dioptric values (-7D to 5D from the camera position at 1D) and pupil sizes (~3mm-8mm).

However, the accuracy of the results will largely depend on the calibration factor, which is often obtained from a sample of mostly Caucasian individuals. Previous studies have demonstrated that ethnic differences (Sravani et al., 2015) and further inter-individual differences (Bharadwaj et al., 2013; Ghahghaei et al., 2019) affect this calibration factor, reducing the accuracy of the results. They have also suggested how a correction factor specific to each individual can be quickly found and used to reduce these errors significantly, that is independent of the viewing distance used To find this individual correction factor we followed the method described by Sravani et al. (2015).

A fixation stimulus illuminated by the green LED is presented at 1D from the participant and viewed monocularly through the opposite eye for which the calibration was being performed.

The eye being calibrated (right eye in experiments 1 and 2, and left eye in experiment 3) was covered by an infrared filter, allowing to measure its refractive state while occluding the stimulus. A series of trial lenses from -4D to 7D in 1D steps were also placed in front of this eye, and refraction was measured binocularly for at least 30 seconds for each of the lenses. This method allows to obtain the defocus measured by the photorefractor for objective values of defocus introduced for the calibrated eye through the trial lenses, while also accounting for the changes in accommodation by concurrently measuring the refraction of the left eye that views the stimulus. An individual correction factor was obtained by plotting the average differences in refraction between the two eyes as a function of the trial lens used and fitting a linear regression through the linear portion of this function. The inverse of the resulting slope was then used to rescale all the refractive data obtained for this participant. An example of the results of the calibration procedure obtained for one subject are shown in Supp Fig 1. In experiment 3, to account for the differences in refractive error between the two eyes, the average refractive difference with no trial lens (0D) was obtained for each participant and subtracted from their data.

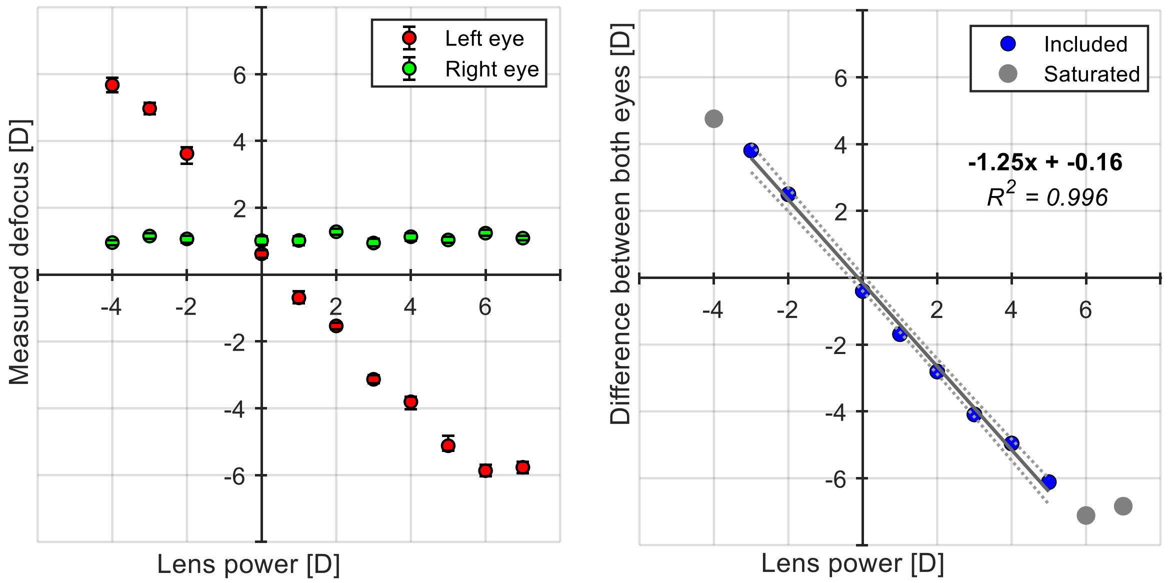

Supp Fig 1. Results of the calibration procedure for one participant. The left panel shows the median and 25th and 75th percentiles of the measured defocus in both eyes as a function of the power of the lens used in front of the left eye. The right eye was uncovered and accommodating on a fixed target, while the left eye was covered by an infrared filter and different lenses. The right panel shows the difference in defocus between both eyes and the fitted linear regression. The steep slope indicates that the photorefractor overestimates the defocus in the left eye of this participant, measuring 1.25 dioptres for each 1 dioptre of real defocus. The inverse of this slope can be used to rescale the refraction measurements and correct the overestimation.

Results.

Determining the accommodative range of observers.

Accommodative range varies widely among individuals, particularly in a sample of observers with differences in age and refractive error. Within the accommodative range the response is expected to be quasi-linear with respect to the demand, while beyond it – that is, for demands higher than the near point of accommodation or lower than the far point – the response becomes saturated as the power of the crystalline lens cannot longer increase or decrease, respectively. The slope of the accommodation response curve is usually assessed within this linear accommodative range; thus, it was important to determine the near and far point of accommodation for each individual observer in our sample.

To do this, we calculated for each participant the gradient of the accommodation response as a function of distance in dioptres for each illuminant. At distances where the gradient dropped by 50% or more when compared to the overall median gradient, the response was determined to be saturated, while the distances where the gradient was maintained were taken to be within the accommodative range or linear portion of the accommodation response curve. This process was done for individual illuminants, such that the response to each could saturate at different distances (due to the differences in accommodative demand caused by LCA). The results of this process were visually inspected and agreed well with the evaluation of the experimenter (see Figure 7 for an example of the results for one subject). In all the analyses presented in following sections, the saturated portion of the accommodation response was omitted (i.e., only accommodative and pupil responses within the linear portion of the accommodation response curve were included), except for section 2.3.6, where some analyses include responses beyond the accommodative range, as detailed there.

Effects of LCA on the accommodation response curve.

Experiment & Illuminant		Slope [D/D]							Intercept [D]			
		Est.	95% CI		RE SD	t-ratio	df	p-value	Est.	RE SD	t-ratio	p-value
1	661nm	0.89	0.81	0.97	0.10				0.33	0.16		
	588nm	0.79	0.74	0.84	0.04	-3.84	1246	*<0.001*	0.36	0.07	0.69	0.488
	527nm	0.79	0.74	0.84	0.03	-4.15	1246	*<0.001*	0.21	0.08	-2.05	*0.041*
	460nm	0.71	0.65	0.77	0.06	-5.90	1246	*<0.001*	0.19	0.19	-1.61	0.107
	441nm	0.63	0.58	0.69	0.05	-9.07	1246	*<0.001*	0.23	0.24	-0.95	0.341
	broadband	0.90	0.84	0.95	0.05	0.08	1246	0.940	0.12	0.09	-3.67	*<0.001*
2	661nm	1.07	0.91	1.22	0.23				-0.16	0.71		
	588nm	0.94	0.88	1.01	0.07	-3.89	3157	*<0.001*	-0.08	0.10	1.43	0.154
	527nm	0.92	0.84	1.00	0.10	-3.66	3157	*<0.001*	-0.21	0.23	-0.53	0.595
	460nm	0.82	0.72	0.91	0.13	-5.07	3157	*<0.001*	-0.21	0.30	-0.46	0.648
	441nm	0.78	0.69	0.86	0.11	-6.59	3157	*<0.001*	-0.21	0.26	-0.49	0.626
	broadband	1.00	0.93	1.07	0.08	-1.86	3157	0.063	-0.30	0.15	-2.02	*0.044*
3	610nm	1.15	0.67	1.64	0.65				-1.16	3.24	-0.95	
	528nm	1.09	0.81	1.36	0.36	-0.49	1774	0.624	-1.13	1.64	0.06	0.956
	459nm	0.95	0.66	1.24	0.38	-1.40	1774	0.161	-0.93	1.84	0.33	0.744
	broadband	1.11	0.92	1.31	0.26	-0.43	1774	0.667	-0.98	1.24	0.38	0.702

*Supp Table 1. Linear mixed models of accommodation as a function of distance in dioptres and illuminant for each experiment: Accommodation ~ Distance * Illuminant + (1 + Distance * Illuminant | ID). The estimated coefficients and their 95% confidence intervals (95% CI) have been used to calculate the estimated slopes (in dioptres/dioptres) and intercepts (in dioptres) for each illuminant. The intercept specifies where the average person accommodates for a stimulus at infinity under the specified illuminant, and the slope specifies how that changes with dioptric distance. The random effects standard deviations (RE SD), t-ratios, degrees of freedom (df), and p-values are also shown. The t-tests compare within each experiment, the slope and intercept estimates of each illuminant with the estimates for the longest-wavelength illuminant.*

Experiment & Contrasted illuminants		Slope pairwise comparisons				Intercept pairwise comparisons			
		diff.	df	t-ratio	p-value	diff.	df	t-ratio	p-value
	red - orange	0.10	3.99	7.68	*0.009*	-0.04	3.63	-1.16	0.997
	red - green	0.10	3.66	7.90	*0.011*	0.11	3.83	3.65	0.299
	red - blue	0.18	4.52	13.70	*<0.001*	0.13	5.35	3.62	0.186
	red - violet	0.26	4.09	24.30	*<0.001*	0.09	5.74	2.44	0.554
	red - white	0.00	4.26	-0.16	1.000	0.20	4.03	6.18	*0.049*
	orange - green	0.00	4.05	0.06	1.000	0.15	4.00	5.18	0.095
	orange - blue	0.08	4.49	9.42	*0.003*	0.17	5.39	5.36	*0.035*
**1**	orange - violet	0.16	4.30	18.57	*<0.001*	0.13	5.79	3.43	0.201
	orange - white	-0.10	3.80	-9.32	*0.005*	0.24	3.75	8.21	*0.023*
	green - blue	0.08	3.88	9.45	*0.005*	0.02	4.66	0.72	1.000
	green - violet	0.16	4.20	32.52	*<0.001*	-0.02	5.43	-0.73	1.000
	green - white	-0.10	3.74	-9.85	*0.005*	0.09	3.90	3.28	0.383
	blue - violet	0.08	4.23	10.98	*0.002*	-0.04	4.97	-1.57	0.947
	blue - white	-0.19	3.72	-18.36	*<0.001*	0.07	4.68	2.29	0.685
	violet - white	-0.26	4.52	-29.51	*<0.001*	0.11	5.74	3.23	0.252
	red - orange	0.12	7.13	3.92	*0.042*	-0.08	6.74	-1.39	0.970
	red - green	0.15	7.57	3.85	*0.042*	0.05	7.60	0.56	1.000
	red - blue	0.25	7.72	5.40	*0.006*	0.05	7.73	0.48	1.000
	red - violet	0.29	7.65	6.99	*0.001*	0.05	7.68	0.52	1.000
	red - white	0.06	7.40	1.93	0.451	0.13	7.23	2.09	0.683
	orange - green	0.03	7.38	0.73	0.972	0.13	7.58	1.47	0.950
	orange - blue	0.13	7.50	3.37	0.078	0.13	7.71	1.24	0.987
**2**	orange - violet	0.17	7.44	4.61	*0.017*	0.13	7.64	1.35	0.974
	orange - white	-0.06	7.17	-1.82	0.510	0.21	7.27	3.10	0.220
	green - blue	0.10	7.12	3.57	0.064	0.00	7.10	0.04	1.000
	green - violet	0.14	7.48	3.98	*0.036*	0.00	7.40	0.02	1.000
	green - white	-0.08	7.36	-2.49	0.238	0.09	7.57	1.06	0.997
	blue - violet	0.04	7.16	1.27	0.794	0.00	6.68	-0.02	1.000
	blue - white	-0.18	7.60	-4.60	*0.017*	0.08	7.77	0.75	1.000
	violet - white	-0.22	7.56	-5.87	*0.004*	0.08	7.76	0.77	1.000
	red - green	0.07	5.84	0.93	0.790	-0.03	5.88	-0.10	1.000
	red - blue	0.21	5.85	2.68	0.128	-0.23	5.90	-0.62	0.992
**3**	red - white	0.04	5.73	0.82	0.845	-0.18	5.83	-0.73	0.984
	green - blue	0.14	5.13	4.46	*0.023*	-0.20	5.29	-1.35	0.797
	green - white	-0.03	5.35	-0.72	0.884	-0.15	5.29	-1.10	0.901
	blue - white	-0.16	5.26	-5.17	*0.012*	0.05	5.35	0.33	1.000

Supp Table 2. Post-hoc pairwise comparisons of the slope and intercept estimates between illuminants. The estimated differences (diff.), degrees of freedom (df), t-ratios and p-values are shown. The shaded p-values in italics represent significant results at the 0.05 level. The different illuminants have been named according to their colour for easier reading of the

results. For experiments 1 and 2, they represent the 661 nm (red), 588 nm (orange), 527 nm (green), 460 nm (blue) and 441 nm (violet) illuminants; while for experiment 3 they represent the 610 nm (red), 528 nm (green) and 459 nm (blue) illuminants.

Results of the linear mixed models of accommodation for individual participants.

In this section, we present additional results of the linear mixed models used on the linear portion of the accommodation response curves, as described in the paper. The table shows the slopes and intercepts estimated for individual participants under each illuminant, which were calculated from the estimated random effects coefficients of the linear mixed models fitted to the data of each experiment.

**subject**	**Experiment 1**					
	broadband	661 nm	588 nm	527 nm	460 nm	441 nm
1	0.89 (0.02)	0.81 (0.02)	0.81 (0.02)	0.79 (0.01)	0.68 (0.02)	0.62 (0.02)
	0.19 (0.02)	*0.55 (0.03)*	*0.43 (0.03)*	*0.30 (0.03)*	*0.37 (0.05)*	*0.41 (0.06)*
2	0.85 (0.02)	0.78 (0.02)	0.73 (0.02)	0.78 (0.01)	0.70 (0.03)	0.65 (0.02)
	0.06 (0.02)	*0.22 (0.03)*	*0.35 (0.03)*	*0.06 (0.03)*	*-0.09 (0.04)*	*-0.17 (0.05)*
3	0.97 (0.02)	0.85 (0.03)	0.81 (0.02)	0.83 (0.02)	0.82 (0.03)	0.64 (0.03)
	-0.05 (0.03)	*0.12 (0.05)*	*0.33 (0.04)*	*0.18 (0.03)*	*-0.05 (0.06)*	*0.27 (0.08)*
4	0.90 (0.02)	1.10 (0.02)	0.82 (0.02)	0.77 (0.02)	0.67 (0.03)	0.59 (0.03)
	0.12 (0.03)	*0.14 (0.04)*	*0.26 (0.03)*	*0.23 (0.03)*	*0.38 (0.06)*	*0.46 (0.08)*
5	0.92 (0.02)	0.94 (0.02)	0.82 (0.02)	0.80 (0.02)	0.75 (0.03)	0.70 (0.02)
	0.12 (0.02)	*0.33 (0.03)*	*0.32 (0.03)*	*0.24 (0.03)*	*0.14 (0.05)*	*0.05 (0.06)*
7	0.87 (0.02)	0.93 (0.03)	0.76 (0.02)	0.80 (0.02)	0.68 (0.03)	0.60 (0.03)
	0.16 (0.03)	*0.34 (0.04)*	*0.40 (0.03)*	*0.16 (0.03)*	*0.18 (0.05)*	*0.25 (0.07)*
8	0.85 (0.02)	0.80 (0.02)	0.77 (0.02)	0.77 (0.02)	0.68 (0.03)	0.65 (0.03)
	0.21 (0.03)	*0.52 (0.03)*	*0.44 (0.03)*	*0.26 (0.03)*	*0.24 (0.05)*	*0.12 (0.07)*
9	0.91 (0.02)	0.95 (0.02)	0.82 (0.02)	0.79 (0.02)	0.70 (0.03)	0.60 (0.03)
	0.17 (0.03)	*0.39 (0.04)*	*0.37 (0.03)*	*0.28 (0.03)*	*0.37 (0.05)*	*0.49 (0.07)*
	**Experiment 2**					
	broadband	661 nm	588 nm	527 nm	460 nm	441 nm
1	1.10 (0.02)	0.84 (0.02)	1.00 (0.01)	0.99 (0.03)	0.95 (0.03)	0.89 (0.03)
	-0.28 (0.05)	*0.55 (0.05)*	*-0.19 (0.02)*	*-0.28 (0.07)*	*-0.33 (0.08)*	*-0.30 (0.08)*
2	0.89 (0.02)	1.20 (0.03)	0.92 (0.01)	0.79 (0.04)	0.62 (0.04)	0.56 (0.04)
	-0.10 (0.06)	*-0.41 (0.07)*	*0.00 (0.03)*	*-0.04 (0.09)*	*-0.04 (0.09)*	*-0.11 (0.09)*
3	0.99 (0.02)	0.90 (0.03)	1.00 (0.01)	0.92 (0.04)	0.86 (0.04)	0.81 (0.04)
	-0.18 (0.06)	*0.24 (0.07)*	*-0.13 (0.03)*	*-0.14 (0.09)*	*-0.20 (0.09)*	*-0.19 (0.09)*

8	1.00 (0.02)	0.92 (0.03)	0.98 (0.01)	0.96 (0.04)	0.91 (0.04)	0.87 (0.04)
	-0.27 (0.06)	*0.30 (0.06)*	*-0.17 (0.03)*	*-0.30 (0.08)*	*-0.42 (0.08)*	*-0.46 (0.08)*
10	0.96 (0.02)	1.20 (0.03)	0.89 (0.01)	0.92 (0.03)	0.78 (0.03)	0.72 (0.04)
	-0.31 (0.05)	*-0.23 (0.05)*	*-0.05 (0.03)*	*-0.37 (0.07)*	*-0.45 (0.07)*	*-0.50 (0.08)*
11	0.92 (0.03)	1.10 (0.03)	0.82 (0.01)	0.90 (0.04)	0.76 (0.04)	0.74 (0.04)
	-0.30 (0.06)	*-1.10 (0.06)*	*0.12 (0.03)*	*-0.26 (0.09)*	*-0.30 (0.09)*	*-0.26 (0.09)*
12	1.10 (0.02)	1.10 (0.03)	0.99 (0.01)	1.10 (0.04)	0.91 (0.04)	0.77 (0.04)
	-0.56 (0.06)	*0.39 (0.07)*	*-0.13 (0.03)*	*-0.51 (0.09)*	*-0.35 (0.09)*	*-0.17 (0.09)*
13	1.00 (0.02)	0.81 (0.03)	0.99 (0.01)	0.96 (0.03)	0.93 (0.03)	0.90 (0.04)
	-0.23 (0.05)	*0.21 (0.05)*	*-0.13 (0.03)*	*-0.22 (0.07)*	*-0.30 (0.07)*	*-0.28 (0.07)*
14	1.00 (0.03)	1.50 (0.03)	0.90 (0.01)	0.76 (0.04)	0.63 (0.04)	0.72 (0.04)
	-0.44 (0.06)	*-1.50 (0.07)*	*-0.09 (0.03)*	*0.22 (0.10)*	*0.47 (0.09)*	*0.35 (0.09)*
	**Experiment 3**					
	broadband	610 nm		528 nm	459 nm	
2	1.00 (0.01)	0.91 (0.03)		0.92 (0.03)	0.82 (0.03)	
	-1.60 (0.08)	*0.44 (0.11)*		*-1.60 (0.10)*	*-1.60 (0.15)*	
8	1.10 (0.01)	0.96 (0.02)		0.92 (0.02)	0.89 (0.03)	
	-1.60 (0.07)	*0.29 (0.10)*		*-1.40 (0.08)*	*-1.70 (0.14)*	
16	0.98 (0.01)	0.99 (0.03)		1.10 (0.02)	0.95 (0.04)	
	-1.40 (0.08)	*-0.66 (0.11)*		*-1.80 (0.09)*	*-1.80 (0.20)*	
17	2.00 (0.03)	2.60 (0.06)		1.80 (0.03)	1.60 (0.07)	
	1.80 (0.16)	*-8.50 (0.31)*		*2.50 (0.17)*	*3.20 (0.33)*	
18	0.87 (0.01)	0.82 (0.03)		0.96 (0.02)	0.85 (0.05)	
	-1.40 (0.09)	*0.22 (0.11)*		*-1.80 (0.11)*	*-1.90 (0.22)*	
19	0.83 (0.01)	0.82 (0.03)		1.10 (0.02)	0.64 (0.04)	
	-1.20 (0.09)	*-0.18 (0.14)*		*-2.40 (0.09)*	*-1.00 (0.18)*	
20	1.00 (0.01)	0.95 (0.04)		0.92 (0.03)	0.91 (0.04)	
	-1.50 (0.08)	*0.20 (0.14)*		*-1.30 (0.11)*	*-1.70 (0.18)*	

Supp Table 3. Individual slopes and intercepts estimated for the linear portion of the accommodation response curves of each participant to each illuminant used in experiments 1, 2 and 3. For each participant, the first row represents the estimated slopes for each illuminant, and the corresponding conditional standard deviations are shown between parentheses; and the second row in italics represents the estimated intercepts for each illuminant and the corresponding conditional standard deviations between parentheses.

Variability in the accommodation responses to narrowband and broadband illuminants.

Experiment	Parameter	Estimate	CI 95%		t-test	df	p-value	RE SD
1 & 2	Intercept	-1.996	-2.21	-1.78	-18.00	4420	*<0.001*	0.39
	460nm	-0.063	-0.11	-0.02	-2.65	4420	*0.008*	0.04
	527nm	-0.173	-0.23	-0.12	-6.04	4420	*<0.001*	0.07
	588nm	-0.348	-0.44	-0.26	-7.35	4420	*<0.001*	0.15
	661nm	-0.301	-0.37	-0.23	-8.88	4420	*<0.001*	0.09
	broadband	-0.172	-0.23	-0.11	-5.44	4420	*<0.001*	0.08
	Accommodation	0.139	0.08	0.20	4.77	4420	*<0.001*	0.10
3	Intercept	-2.016	-2.18	-1.86	-24.61	2190	*<0.001*	0.25
	528nm	-0.149	-0.22	-0.07	-3.95	2190	*<0.001*	0.10
	610nm	-0.172	-0.26	-0.08	-3.74	2190	*<0.001*	0.13
	broadband	-0.111	-0.17	-0.05	-3.57	2190	*<0.001*	0.08
	Accommodation	0.175	0.10	0.25	4.36	2190	*<0.001*	0.12

Supp Table 4. Linear mixed model results of the logarithmically transformed root-mean-square errors (RMSEs) of an unconstrained linear fit through the within-trial accommodation response, as a function of accommodation and illuminant, in experiments 1 and 2, and experiment 3: log(RMSE) ~ Accommodation + Illuminant + (1 + Accommodation + Illuminant | ID). Coefficient estimates and their 95% confidence interval (CI 95%) are shown, as well as degrees of freedom (df), t-ratios p-values, and the random effects standard deviation (RE SD). The intercepts of the models are the corresponding shortest wavelength illuminants (441nm in experiments 1 and 2, and 459nm in experiment 3) at zero dioptres of accommodation. To interpret the parameter estimates, they must be exponentiated.

Accommodation and pupil size.

Experiment	Parameters	Estimate	CI 95%		t-ratio	df	p-value
1	Intercept	6.11	5.79	6.42	38.09	1448	<0.001
	460nm	0.22	0.11	0.33	3.95	1448	<0.001
	527nm	0.97	0.67	1.27	6.34	1448	<0.001
	588nm	1.66	1.39	1.94	11.98	1448	<0.001
	661nm	1.70	1.38	2.01	10.57	1448	<0.001
	broadband	0.86	0.62	1.10	7.12	1448	<0.001
	Accommodation	-0.75	-0.89	-0.62	-10.81	1448	<0.001
2	Intercept	5.28	4.80	5.76	21.68	3494	<0.001
	460nm	0.23	0.16	0.30	6.22	3494	<0.001
	527nm	0.75	0.59	0.90	9.48	3494	<0.001
	588nm	1.40	1.21	1.58	14.67	3494	<0.001
	661nm	1.26	1.11	1.41	16.22	3494	<0.001
	broadband	0.70	0.55	0.86	8.82	3494	<0.001
	Accommodation	-0.16	-0.26	-0.06	-3.12	3494	0.002
3	Intercept	5.63	5.12	6.15	21.62	912	<0.001
	528nm	0.30	0.18	0.42	4.81	912	<0.001

	610nm	0.45	0.30	0.59	6.06	912	<0.001
	broadband	0.23	0.14	0.31	5.18	912	<0.001
	Accommodation	-0.18	-0.29	-0.06	-3.02	912	0.003

Supp Table 5. Linear mixed model results of pupil diameter for experiments 1, 2 and 3, fitting Pupil Diameter ~ Accommodation + Illuminant + (1 + Accommodation + Illuminant | ID). Coefficient estimates, their 95% confidence interval (CI 95%) and standard errors (SE) are shown, as well as degrees of freedom (df), t-ratios and p-values. The intercepts of the models are the corresponding shortest wavelength illuminants (441nm in experiments 1 and 2, and 459nm in experiment 3) at zero dioptres of refraction.

Accommodative error and visual acuity.

**Visual acuity [logMAR] - Linear portion of the accommodation function**							
**Parameters**	**Estimate**	**CI 95%**		**t-ratio**	**df**	**p-value**	**RE SD**
Intercept	0.04	-0.06	0.15	0.79	8.29	0.452	0.16
Error magnitude [D]	0.10	-0.02	0.21	1.69	8.66	0.126	0.17
Positive sign	0.01	-0.07	0.09	0.26	7.04	0.800	0.11
Illuminant: 528nm	***-0.11***	***-0.17***	***-0.06***	***-3.95***	***8.72***	***0.004***	***0.08***
Illuminant: 610nm	***-0.08***	***-0.14***	***-0.02***	***-2.72***	***8.70***	***0.024***	***0.08***
Illuminant: broadband	***-0.09***	***-0.12***	***-0.05***	***-4.53***	***9.09***	***0.001***	***0.05***
Error magnitude [D] * Positive sign	0.02	-0.10	0.14	0.31	9.70	0.761	0.16

Supp Table 6. Linear mixed model results of visual acuity over the linear portion of the accommodation response curve, as a function of illuminant, accommodative error magnitude, accommodative error sign, and their interaction. The coefficient estimates, their 95% confidence intervals (CI 95%), t-ratios, degrees of freedom (df), p-values, and random effects standard deviations (RE SD) are shown.

**Visual acuity [logMAR] - Overaccommodation**							
**Parameters**	**Estimate**	**CI 95%**		**df**	**t-ratio**	**p-value**	**RE SD**
Intercept *(4mm, 0D, 459nm)*	0.13	-0.06	0.32	4.52	1.31	0.252	0.29
Error magnitude [D]	0.21	0.10	0.31	4.38	3.84	0.016	0.14
Pupil Diameter [mm]	-0.07	-0.15	0.01	5.80	-1.80	0.123	0.10
Illuminant: 528nm	-0.05	-0.11	0.01	10.09	-1.67	0.126	0.08
Illuminant: 610nm	-0.01	-0.08	0.06	9.62	-0.23	0.822	0.09
Illuminant: broadband	-0.04	-0.09	0.01	13.18	-1.68	0.116	0.05

Error magnitude [D] * Pupil Diameter [mm]	***0.08***	***0.02***	***0.13***	***5.39***	***2.78***	***0.036***	***0.07***
**Visual acuity [logMAR] - Underaccommodation**							
**Parameters**	**Estimate**	**CI 95%**		**df**	**t-ratio**	**p-value**	**RE SD**
Intercept *(4mm, 0D, 459nm)*	0.14	0.04	0.25	5.08	2.61	*0.047*	0.14
Error magnitude [D]	0.06	0.00	0.12	9.97	1.82	0.098	0.07
Pupil Diameter [mm]	-0.05	-0.12	0.02	7.09	-1.44	0.192	0.09
Illuminant: 528nm	-0.07	-0.14	-0.01	7.31	-2.16	0.066	0.08
Illuminant: 610nm	-0.04	-0.11	0.03	7.48	-1.21	0.264	0.09
Illuminant: broadband	-0.07	-0.14	-0.01	4.83	-2.15	0.086	0.08
Error magnitude [D] * Pupil Diameter [mm]	0.04	-0.02	0.11	3.53	1.31	0.268	0.09

Supp Table 7. Linear mixed models' results of visual acuity for positive accommodative errors (top) and negative accommodative errors (bottom), as a function of error magnitude, pupil diameter, their interaction, and illuminant. The coefficient estimates, their 95% confidence intervals (CI 95%), t-ratios, degrees of freedom (df), p-values, and random effects standard deviations (RE SD) are shown.

Accommodation response curves for individual participants.

In this section, we present the accommodation response curves of individual participants in experiments 1, 2 and 3. The plots in Supp Fig 2-4 present the accommodation response as a function of the distance in dioptres.

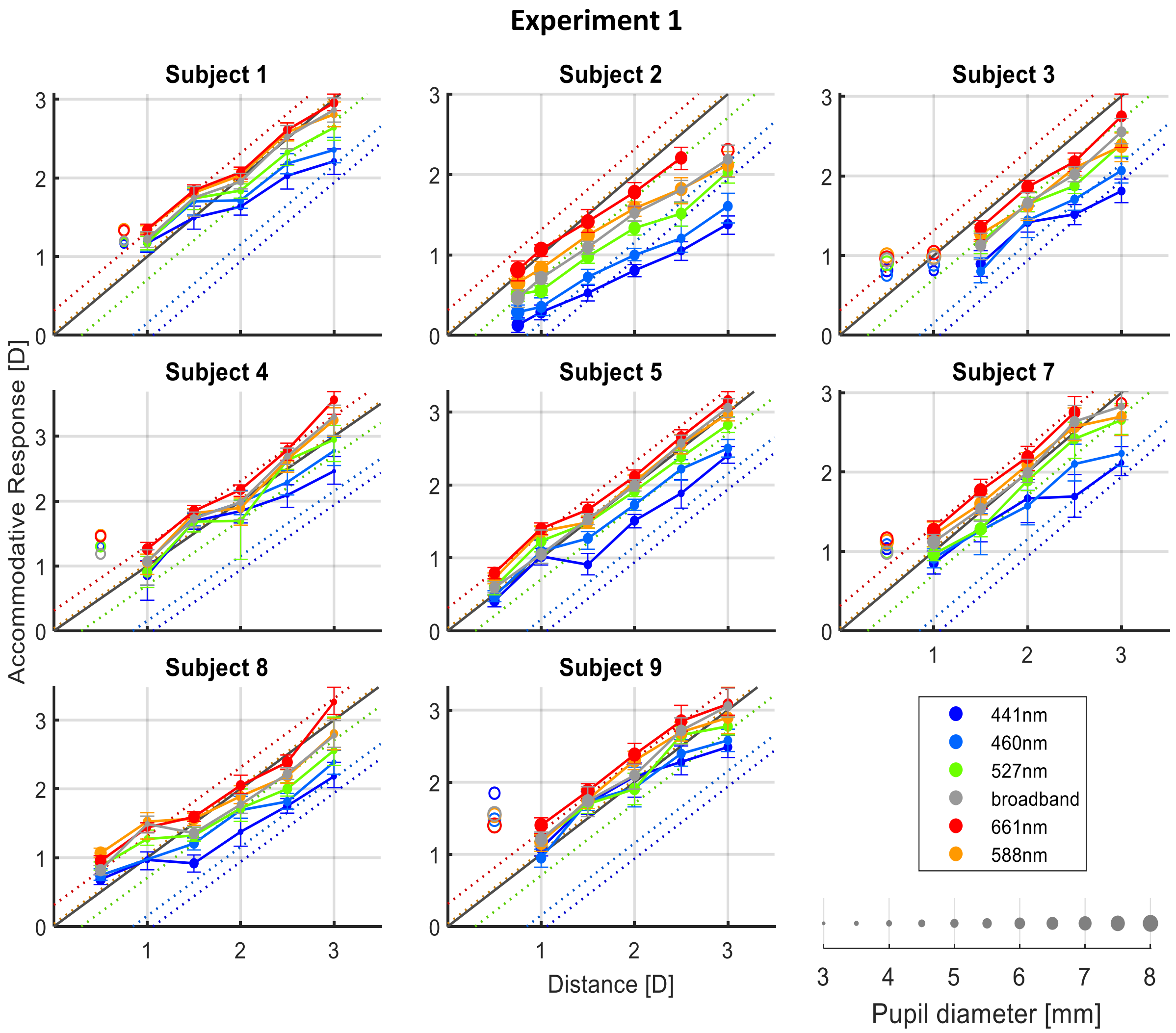

Supp Fig 2. Median accommodation response as a function of distance for individual participants of experiment 1. Each colour represents one illuminant, and the marker sizes represent the median pupil size for each distance and illuminant. The error bars represent the 25th and 75th percentiles of the accommodation response. The filled markers and continuous lines represent the portion of the response curve deemed to be linear, while the unconnected open markers with no error bars represent the portions of the curve identified as saturated. The continuous grey line represents the one-to-one response, while the dotted coloured lines represent the one-to-one response corrected by the LCA defocus for the peak wavelength of each illuminant.

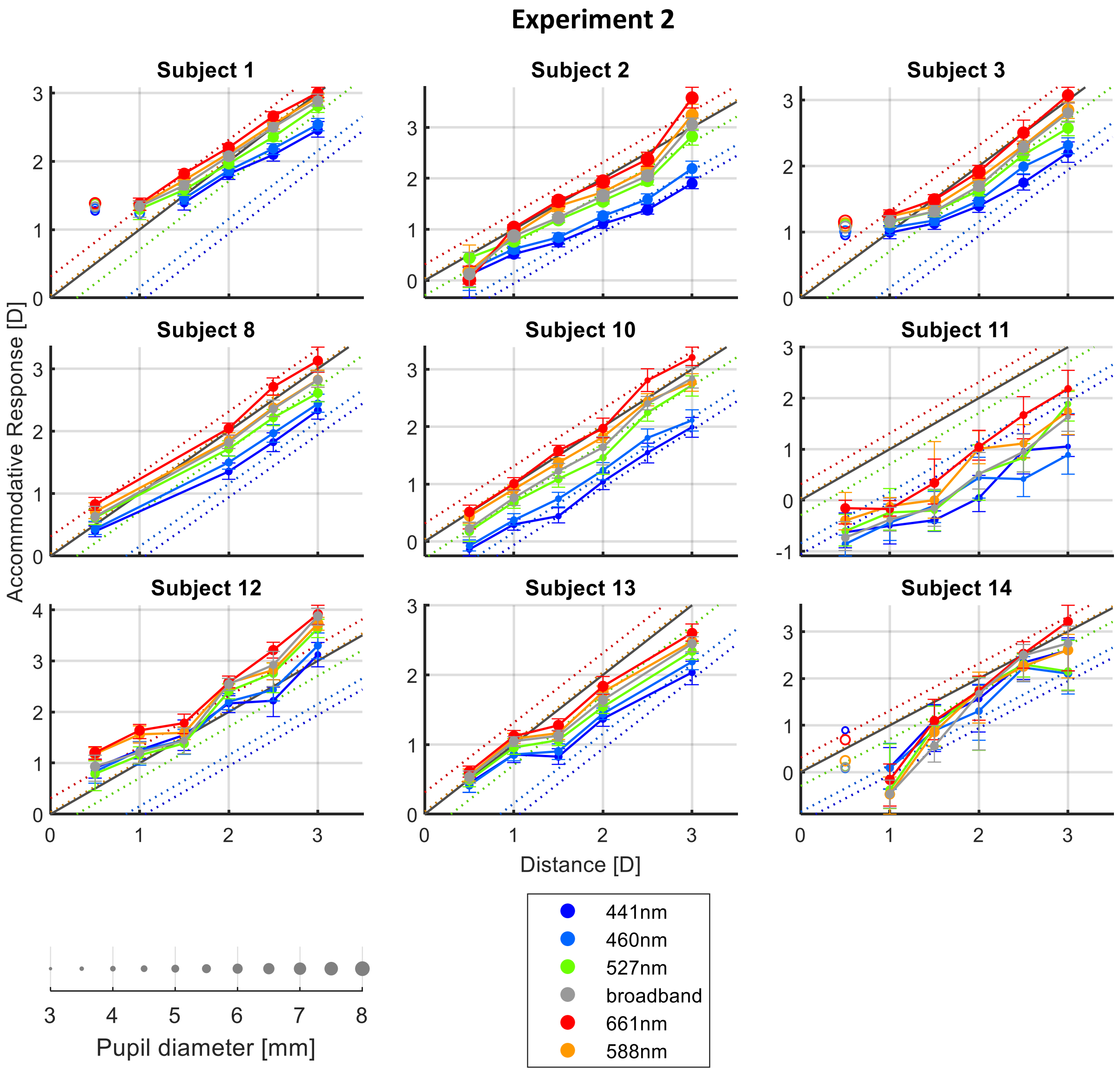

Supp Fig 3. Median accommodation response as a function of distance for individual participants of experiment 2. Each colour represents one illuminant, and the marker sizes represent the median pupil size for each distance and illuminant. The error bars represent the 25th and 75th percentiles of the accommodation response. The filled markers and continuous lines represent the portion of the response curve deemed to be linear, while the unconnected open markers with no error bars represent the portions of the curve identified as saturated. The continuous grey line represents the one-to-one response, while the dotted coloured lines represent the one-to-one response corrected by the LCA defocus for the peak wavelength of each illuminant.

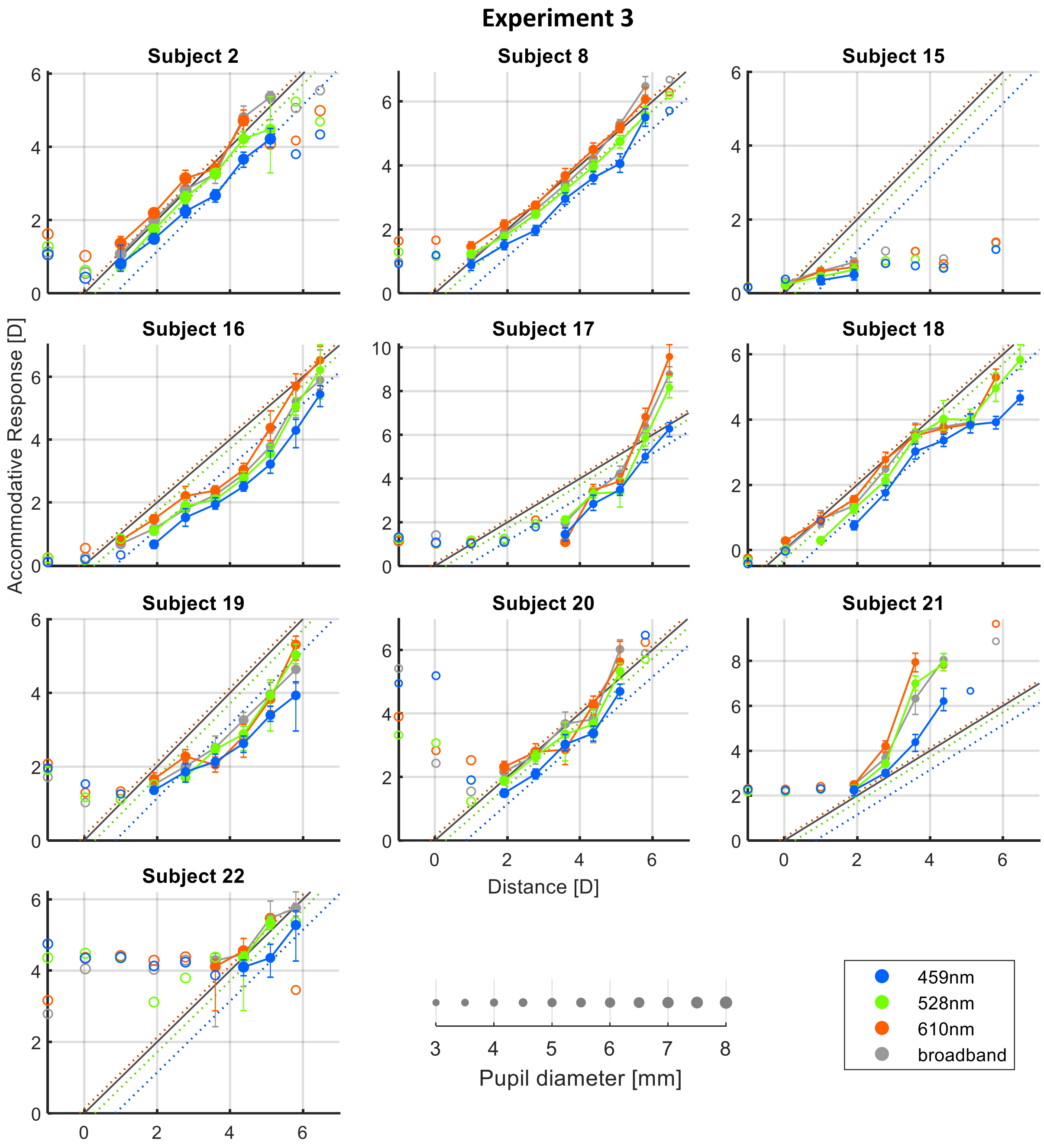

Supp Fig 4. Median accommodation response as a function of distance for individual participants of experiment 3. Each colour represents one illuminant, and the marker sizes represent the median pupil size for each distance and illuminant. The error bars represent the 25th and 75th percentiles of the accommodation response. The filled markers and continuous lines represent the portion of the response curve deemed to be linear, while the unconnected open markers with no error bars represent the portions of the curve identified as saturated. The continuous grey line represents the one-to-one response, while the dotted coloured lines represent the one-to-one response corrected by the LCA defocus for the peak wavelength of each illuminant.

136

Visual acuity results for individual participants.

In Supp Fig 5, visual acuity as a function of accommodative error is plotted for individual participants, as described in the manuscript.

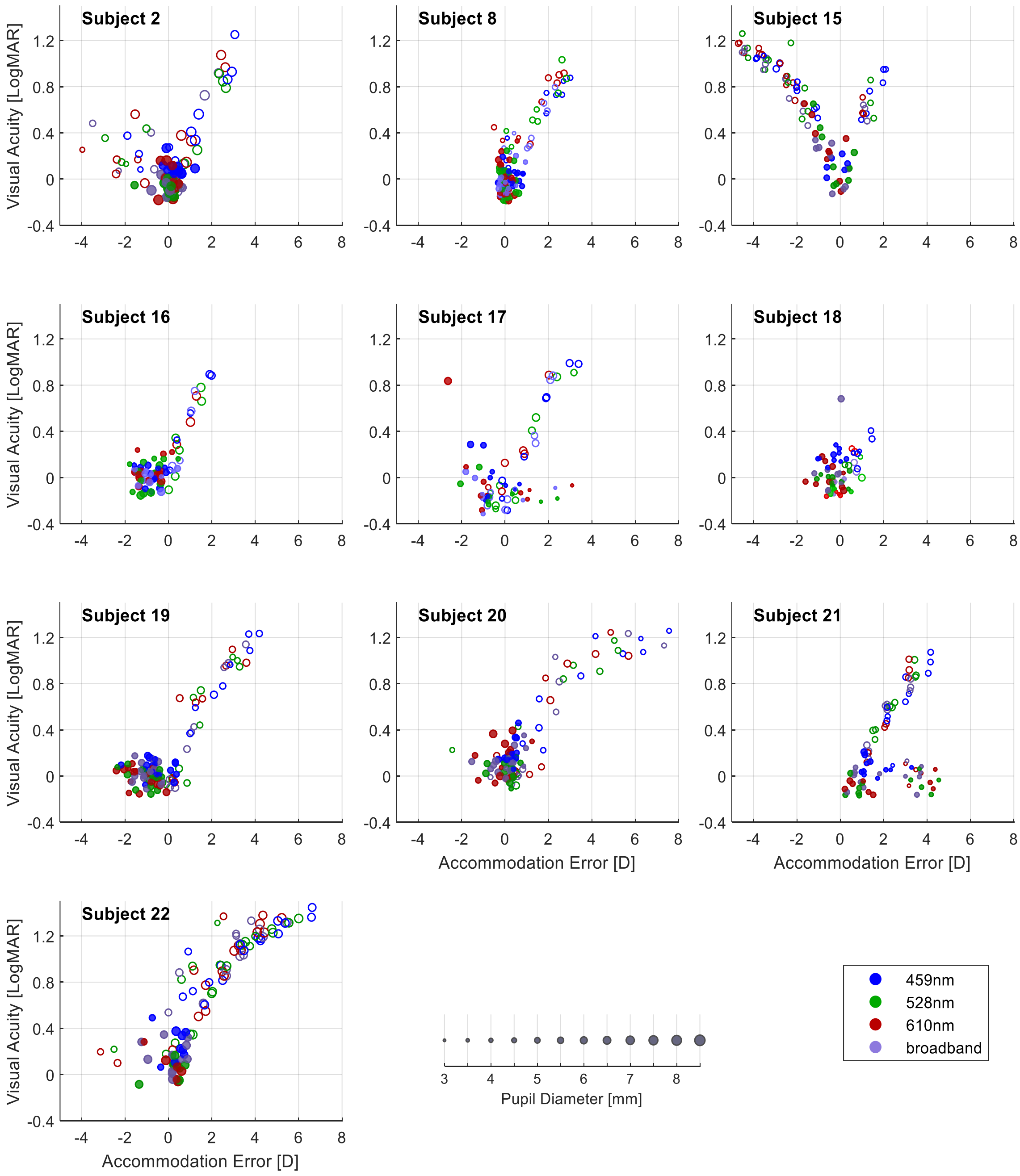

Supp Fig 5. Visual acuity (VA) as a function of the median accommodative error for individual participants of experiment 3. Accommodative error is calculated as the accommodative demand subtracted from the accommodative response. Filled markers correspond to VA measurements over the linear portion of the accommodation response curve, while open markers correspond to measurements at distances where the accommodation function was saturated. The marker colours represent the illuminant used, and the marker sizes the corresponding median pupil size.
